# Distinct types of dendritic Ca^2+^ spikes in CA3 pyramidal neurons associated with neuronal activity changes during navigation

**DOI:** 10.1126/sciadv.aec6015

**Published:** 2026-08-14

**Authors:** Snezana Raus Balind, Balázs Lükő, Imola Plangár, Martin Blazsek, Balázs B. Ujfalussy, Judit K. Makara

**Affiliations:** ^1^ Laboratory of Neuronal Signaling, HUN-REN Institute of Experimental Medicine, Budapest 1083, Hungary.; ^2^ Laboratory of Biological Computation, HUN-REN Institute of Experimental Medicine, Budapest 1083, Hungary.; ^3^ Roska Tamás Doctoral School of Sciences and Technology, Faculty of Information Technology and Bionics, Pázmány Péter Catholic University, Budapest 1083, Hungary.

## Abstract

Active dendrites enrich single-neuron computations by performing nonlinear input integration, including generation of dendritic spikes. Diverse dendritic spike types have been found in various cortical neuron classes in vitro; however, their in vivo prevalence and roles remained elusive. We measured calcium activity in apical dendrites and soma of pyramidal cells in the hippocampal area CA3 (CA3PCs) during virtual navigation in mice. Although dendritic activity was generally synchronous with the soma, their correlation decreased with dendritic distance. We identified two types of regenerative dendritic activities: slow, large-amplitude global Ca^2+^ events representing putative Ca^2+^ plateaus and fast Ca^2+^ events with large dendrite-to-soma attenuation, representing putative dendritically initiated Ca^2+^ spikes. Ca^2+^ plateaus contributed to spatially tuned activity but only occasionally induced new place fields, suggesting conditional induction of synaptic plasticity. In contrast, fast Ca^2+^ spikes were often followed by transient increase in somatic activity without spatial tuning, suggesting increased excitatory inputs or excitability. Our results unveil previously unidentified mechanisms whereby dendritic activity shapes output of CA3PCs in vivo.

## INTRODUCTION

Feature selective firing of hippocampal pyramidal cells (PCs), such as environment- and location-specific activity during spatial navigation, is thought to be the basic substrate of hippocampal spatial maps and episodic memory. A longstanding aim is to understand the cellular mechanisms that determine the activity of individual hippocampal PCs in a given environment. While synaptic inputs and somatic excitability have been assumed to be primary factors, active dendritic integration could also have a contribution (*1*). In vitro experimental work (*2*–*5*) demonstrated that PCs are equipped with multifaceted dendritic mechanisms to perform complex nonlinear processing of spatiotemporally patterned synaptic inputs arriving onto the extensively branching dendritic tree. These include robust modulation of the amplitude and kinetics of postsynaptic voltage responses by regenerative dendritic spikes generated by the activation of dendritic voltage-dependent ion channels. In acute slices, different types of dendritic spikes occur in compartmentalized and global forms (*1*, *6*, *7*) and shape neuronal output in different ways. Local Na^+^ and *N*-methyl-d-aspartate (NMDA) receptor–mediated spikes are prevalent in thin terminal dendrites, and they can produce precisely timed somatic action potentials (APs) or simple burst firing, respectively (*2*, *3*, *8*). In contrast, combined Ca^2+^ and NMDA spikes (also called dendritic plateau potentials) are typically global long-duration dendritic spikes that are thought to be generated in distal apical dendrites and produce characteristic complex spike burst (CSB) firing at the soma (*4*, *9*–*12*). In addition to their instantaneous effect on somatic output, dendritic spikes can also induce synaptic plasticity even upon a single or few occurrences (*13*–*16*). Altogether, due to their theoretical advantages and robust effects in vitro, dendritic spikes have been widely proposed to strongly shape neuronal activity patterns and computations.

Despite these predictions, in vivo experiments—catapulted in the past 15 years by developments in Ca^2+^ (and more recently voltage) imaging tools applicable in behaving animals—so far found relatively modest prevalence and functional roles of local dendritic activity in PCs in awake behaving animals. Studies in various cortical areas showed that the activity in PC dendrites is generally well coupled with that of the soma with relatively rare compartmentalized dendritic signals [(*11*, *17*–*25*) but see (*26*)], although in some cell types local dendritic activity could be linked to behaviorally relevant variables (*17*–*19*, *22*, *27*). Instead, the focus has shifted onto global Ca^2+^ plateaus, which could be detected across different PC types, and were found to contribute to behavioral performance in tasks including sensory perception and active sensing (*28*, *29*). Work in the hippocampus, focusing primarily on PCs of the CA1 area (CA1PCs), has also begun to explore the roles of dendrites in defining spatially tuned somatic firing properties [i.e., place fields (PFs)] during navigation in head-fixed mice. Ca^2+^ imaging in perisomatic dendrites of CA1PCs showed that their activity dominantly coincides with that of the soma. Localized dendritic signals were observed relatively rarely; nevertheless, the incidence of local activity depended on dendrite location (apical versus basal), novelty, learning, and brain state (*30*–*33*), and local dendritic activity could precede future PFs (*30*). More prominent roles have been shown for global dendritic Ca^2+^ plateaus, which were associated with PF prevalence and stability (*30*, *33*), and most notably, have been found to initiate sudden formation of new PFs even in familiar environments (*34*–*36*) by inducing a non-Hebbian form of plasticity of glutamatergic synapses with a seconds-long temporal coincidence window [called behavioral timescale synaptic plasticity or BTSP (*14*, *37*)]. These results suggest that Ca^2+^ plateaus may play a fundamental role in creating and shaping hippocampal cognitive maps. However, the efficacy and prevalence of natural Ca^2+^ plateau–mediated PF formation remained incompletely clarified (*38*, *39*). Furthermore, similar investigations are essential in other hippocampal principal cell types to determine whether the forms, prevalence, and functional roles of regenerative dendritic activities are ubiquitous or cell type- or circuit-specific.

A particularly relevant cell type for comparison are the PCs of the CA3 area (CA3PCs), which have different morphological and electrophysiological properties and operate in a circuit with different connectivity and computational properties from those of CA1PCs. In acute slices, dendrites of CA3PCs exhibit qualitative and quantitative differences in dendritic spike properties compared to those in CA1PCs (*5*, *40*, *41*). In particular, their apical dendritic trunks (which are more numerous than those of CA1PCs) can generate two different Ca^2+^spike types: a slow type that is activated by backpropagating APs (bAPs) and can produce prolonged somatic CSB firing; and another, unusually fast, hybrid Ca^2+^/Na^+^ spike type that is initiated in distal apical dendrites and can produce single APs or short bursts at the soma (*40*, *42*). Furthermore, cholinergic modulation prolongs the kinetics of Ca^2+^ spikes, facilitating CSBs (*42*). These in vitro results raise the possibility of diverse computations whereby dendrites can shape neuronal output, modify tuning, and support plasticity in CA3PCs. However, the relationship of dendritic and somatic activities and the impact of different dendritic spike types on the plasticity of tuning properties of CA3PCs in vivo have so far remained largely unexplored. A recent study (*43*) proposed that CSBs—presumably produced by global Ca^2+^ plateaus (*10*, *40*) —can induce new PFs in CA3PCs similar to BTSP in CA1PCs, albeit with a different temporal kernel; however, they observed only a few spontaneous PF formation events and mostly studied PF induction by strong depolarizing somatic current injections.

Here, we measured Ca^2+^ activity quasi-synchronously in the apical dendrites and soma of individual CA3PCs in mice navigating in virtual environments. Although dendritic activity was generally synchronous with the soma, we identified two types of somatodendritic Ca^2+^ activities that could be explained by regenerative dendritic spikes: slow, large-amplitude global Ca^2+^ events representing putative Ca^2+^ plateaus and fast-decaying Ca^2+^ events with high dendrite/soma amplitude ratio (AR) and limited propagation representing putative dendritically initiated fast Ca^2+^ spikes. We found that Ca^2+^ plateaus contributed to spatially tuned activity of CA3PCs and on few occasions could induce new PFs via a mechanism consistent with CA3 BTSP rules (*43*). In contrast, fast Ca^2+^ spikes were often followed by a temporary increase in somatic activity without spatial tuning. Our results indicate that different types of regenerative dendritic activities are accompanied by distinct changes in the output of CA3PCs in vivo.

## RESULTS

### Ca^2+^ imaging in PCs of the CA3a area during virtual navigation

**Fig. 1.**
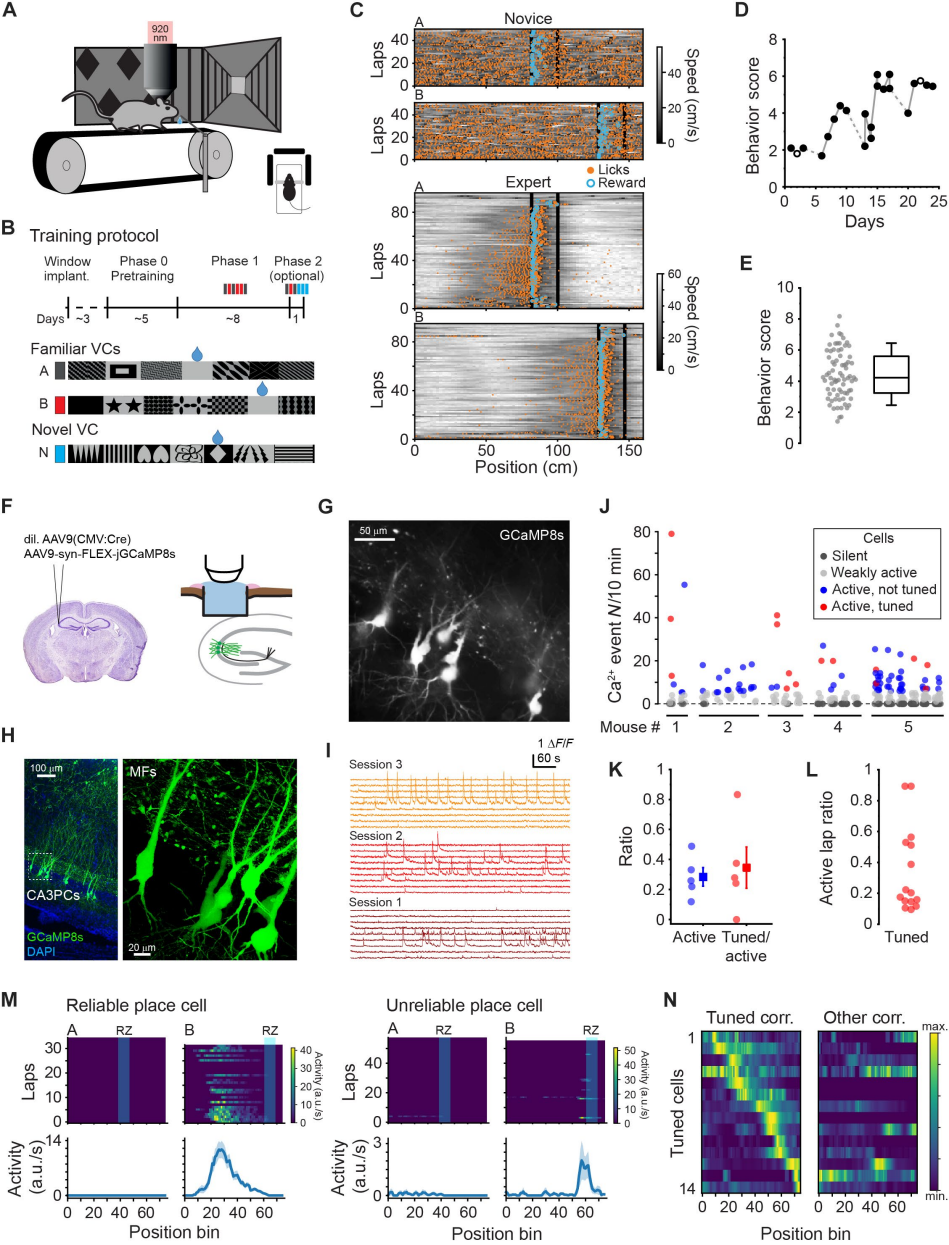
Ca^2+^ activity of CA3PCs during virtual navigation. (**A**) Behavioral setup. (**B**) Experimental timeline (top) and VCs (bottom). (**C**) Lap-by-lap behavior of an example mouse at training start (novice) and after learning (expert). (**D**) Learning curve of the mouse in (C) [open circles: sessions in (C); dashed lines: weekends without training; some days contained two sessions]. (**E**) Behavior scores in 76 imaging sessions (17 mice). Box plot: median (central line), 25th to 75th percentiles (box), and 10th to 90th percentiles (whiskers). (**F**) Virus injection (left; image from Allen Brain Atlas) and imaging (right) procedures. (**G**) Example in vivo two-photon (2P) imaging field of view (FOV; cropped) with moderately densely labeled CA3PCs. (**H**) Post hoc confocal image of a hippocampal section from the mouse in (G), stained for 4′,6-diamidino-2-phenylindole (DAPI; blue). Note thorny excrescences on apical dendrites of CA3PCs and labeled mossy fibers (right). MFs, mossy fibers. (**I**) GCaMP8s-mediated somatic Ca^2+^ signals from the mouse in (G). (**J**) Activity (Ca^2+^ event rate) measured in densely labeled CA3PCs (dots) per sessions (columns) in five mice. Colors: silent (dark gray; 0 events/10 min), weakly active (light gray; <5 events/ 10 min), active (>5 events/10 min) untuned (blue), and spatially tuned (red) cells. (**K**) Ratios of active/all labeled cells (blue) and tuned/active cells (*n* = 5 mice). (**L**) Ratio of active laps in the PF of tuned cells (*n* = 14 cells from five mice with dense labeling; one cell was tuned in two sessions). (**M**) Two example tuned CA3PCs with high (left; reliable) and low (right; unreliable) active lap ratio. Top: Lap-by-lap activity (inferred spike rate) in the two VCs. Bottom: Averaged tuning curves (mean ± SEM). a.u., arbitrary units. (**N**) Spatial tuning map of 14 cells in the VC where they were tuned (left; if tuned in both, the VC with higher spatial information) and in the other VC (right), both sorted according to the position of the tuning curve peak in the tuned VC. max., maximum; min., minimum; corr., corridor.

To study the dynamics of somatic and dendritic activity in CA3PCs during goal-oriented navigation, we developed a behavioral paradigm where water-restricted, head-fixed FVB/AntJ mice were trained to run in a virtual reality system including a treadmill surrounded by monitors displaying virtual corridors (VCs; Fig. 1A). Mice learned to navigate in two pseudorandomly presented, 160-cm-long VCs (VC_A_ and VC_B_) with distinct wall patterns and to collect water reward in an 18-cm-long reward zone (RZ) that was located at different distances in the two VCs but marked with the same wall cue (Fig. 1B). Behavioral parameters (speed dynamics and lick rate) showed that over the course of the ∼2 weeks of training, mice gradually learned to discriminate between the two VCs and identified the location of the RZs where they slowed down and exhibited anticipatory licking (Fig. 1C). The learning process was evaluated by computing a behavioral score (Fig. 1, D and E), which included the ratio of correct choices (licking in the RZ) and various parameters of corridor-specific anticipatory behavior (see Materials and Methods). A subset of mice (*n* = 5) with expert behavior in the two familiar VCs was also exposed to a novel VC (with different wall pattern and RZ at an intermediate location without the familiar RZ cue; Fig. 1B) in which their behavior was typically disrupted, as expected (fig. S1, A and B).

The highly sensitive genetically encoded Ca^2+^ indicator GCaMP8s (*44*) was expressed in the left dorsal hippocampal CA3a area at various densities using different dilutions of two adeno-associated viruses (AAVs) delivering Cre recombinase and floxed GCaMP8s (see Materials and Methods), similar to strategies in previous studies (*33*, *45*). The activity of CA3PCs was monitored using in vivo Ca^2+^ imaging through an implanted imaging cannula placed above the virus-infected CA3 area with the overlying cortex removed (Fig. 1F). The CA3 region was identified on the basis of either the presence of thorny excrescences on the apical dendrites of PCs or on the presence of mossy fibers in stratum lucidum (Fig. 1, G and H, and fig. S1C), which were typically also labeled (fig. S1C).

In mice with moderate to dense labeling (somata of ∼6 to 40 CA3PCs visible in a single focal plane; Fig. 1G), we first evaluated the somatic activity dynamics of CA3PCs imaged with GCaMP8s during the task. Active cells (defined as ≥5 detected Ca^2+^ events/10 min; fig. S1D) constituted a variable fraction of imaged CA3PCs among different animals (mean ± SEM: 28.3 ± 6.2%; *n* = 5 mice, two to six sessions averaged per mouse; Fig. 1, I to K). A subset of the active CA3PCs (34.6 ± 13.7%; *n* = 5 mice) exhibited spatially tuned activity in at least one of the VCs (Fig. 1, K and M), although the lap-to-lap reliability of activity was heterogeneous among cells (Fig. 1, L and M). Tuned cells discriminated the two VCs, as expected (Fig. 1, M and N). Similar results were obtained when CA3PC activity was monitored in Thy1-GCaMP6s mice (C57BL/6J background; *n* = 6), where we could image ∼10 times larger number of cells allowing more reliable estimations. The ratio of active cells was higher in the transgenic mice (fig. S1, E and F), which may, in part, be due to differences in mouse strain, Ca^2+^ sensor type, or expression strategy. Altogether, these results indicate that CA3PCs exhibit sparse spatially tuned activity that can be monitored using Ca^2+^ imaging with GCaMP8s in head-fixed mice navigating in virtual environments in our paradigm, akin to similar studies (*46*, *47*).

### Distance-dependent correlation of somatic and apical dendritic activity

**Fig. 2.**
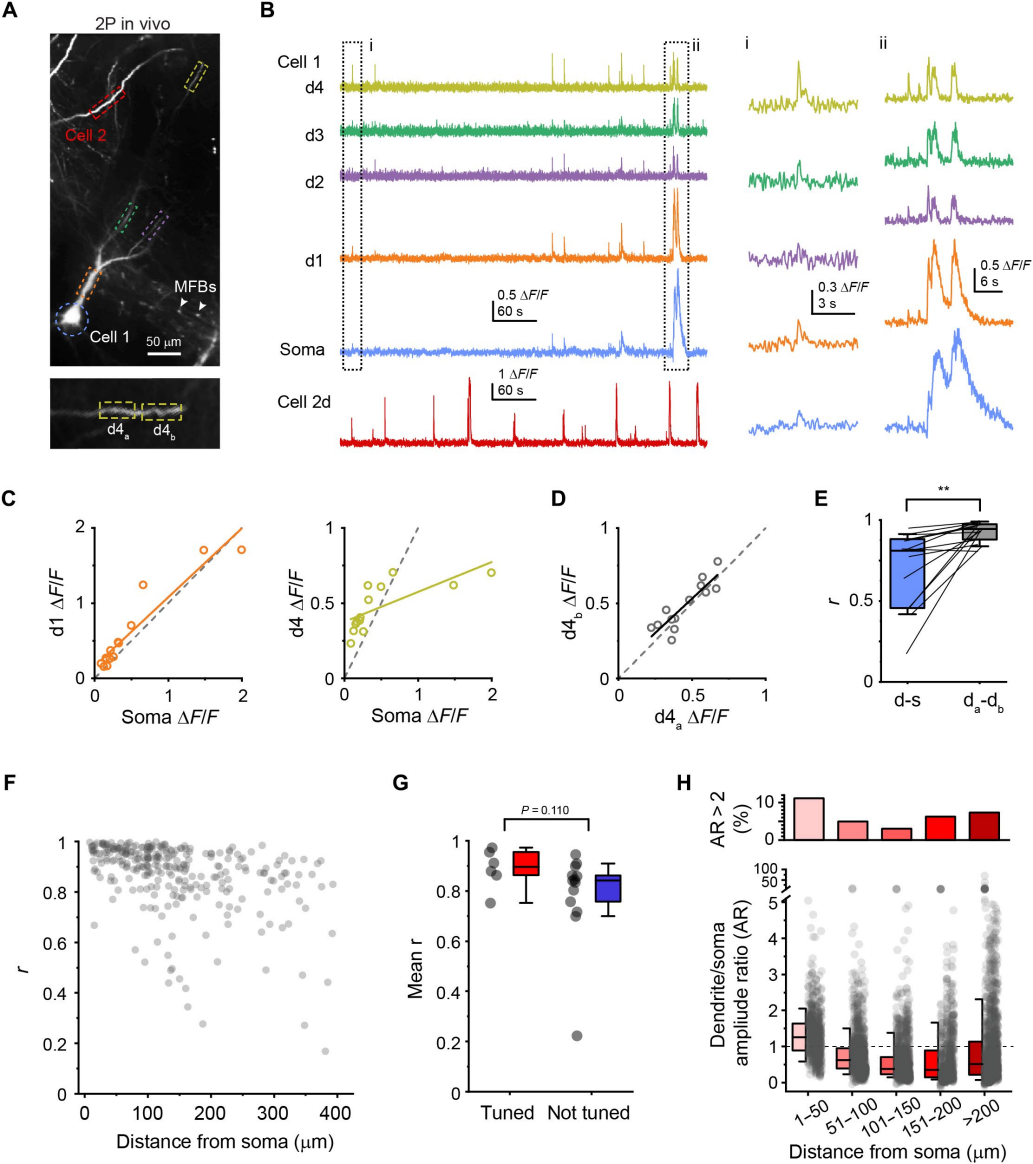
Distance-dependent correlation of somatodendritic activity. (**A**) In vivo 2P image of a GCaMP8s-expressing CA3PC (cell 1). Colored boxes label regions of interest (ROIs) analyzed in (B) (see ROI contours in fig. S2F). Note large mossy fiber boutons (MFBs; arrowheads). Red box indicates dendrite of another cell. Bottom: Distal dendritic segment (yellow box in top panel) split to two neighbor ROIs [see (D)]. (**B**) Ca^2+^ signals measured in the ROIs indicated in (A) (same color code). Note that activities of cells 1 and 2 are independent. Segments in dashed boxes (i and ii) are enlarged on the right. (**C**) Representative correlation plots of Ca^2+^ event amplitudes in two dendrites and the soma. Left: Dendrite 1 (d1). Right: d4 from (A) and (B). Dashed line represents unity; colored straight line indicates regression line. (**D**) Correlation of Ca^2+^ event amplitudes between neighboring ROIs (a and b) on d4 in (A) and (B). Black straight line indicates regression line. (**E**) Pearson’s correlation coefficients of dendrite-soma ROI pairs and neighbor dendrite-dendrite ROI pairs [as in (D)] in cells with long dendritic segment in the focal plane (*n* = 14; Wilcoxon test: *P* = 0.001). d, dendrite; s, soma. (**F**) Correlation coefficients for dendrite-soma Ca^2+^ signal amplitudes as a function of dendritic ROI distance from soma (*n* = 290 ROI pairs in 23 cells; Spearman *R* = –0.498, *P* < 0.001; see also fig. S3, A and B, for data per cell). (**G**) Mean correlation coefficient of distal dendrite-soma ROI pairs in cells with spatially tuned (*n* = 6 cells in five mice) or untuned (*n* = 15 cells in 12 mice) activity in the session. Mann-Whitney test; *P* = 0.110. (**H**) Distance dependence of dendrite-soma Ca^2+^ event peak AR (pooled from 31 cells; event *N* in individual bins: 727 to 1493; see fig. S3G for data per cell). Kruskal-Wallis test: *P* < 0.001. Top; Fraction of events with AR > 2. Different bins contain data mixed from both the same and different cells (fig. S3G).

We next sought to determine the Ca^2+^ activity dynamics of apical dendrites and their relationship with somatic activity. Exploiting the near-horizontal orientation of PCs in distal CA3, we imaged in one or two (relatively close) focal planes (Fig. 2A and fig. S2A) to quasi-simultaneously capture Ca^2+^ activity in the soma and multiple segments of the apical dendritic arbor (Fig. 2B and fig. S2B). Using diluted Cre recombinase to yield 1 to 10 labeled PC somata in a field of view (FOV) with ∼300- to 550-μm-long sides allowed us to match dendrites to their parent somata based on structural and functional identification due to the unique three-dimensional (3D) architecture of each apical tree, the relatively sparse activity and the occurrence of large-amplitude global correlated Ca^2+^ events in most cells (see below, fig. S2, C and D, and Materials and Methods). All neurons included in our study were confirmed to be CA3PCs based either on their depth and orientation, the presence of thorny excrescences on their apical dendrites (visualized either in vivo or by post hoc confocal microscopy in vitro; fig. S2E), and/or the presence of labeled mossy fibers in str. lucidum. Imaging 34 active cells for 10 to 25 min per session in 15 mice (one to nine sessions per animal) performing the task at variable stages of proficiency, we collected Ca^2+^ transients in dendritic segments at different distances up to ∼400 μm from the soma.

Ca^2+^ transients detected in the somata and connected apical dendrites demonstrated overall highly correlated activity: Events appearing in a region of interest (ROI) were usually accompanied by events in several or all other ROIs, including the soma (Fig. 2B and fig. S2, A to D). Consistent with previous in vitro and in vivo Ca^2+^ measurements in other PC types, Ca^2+^ transients in the soma had generally slower rise and decay kinetics compared to those recorded in their dendritic counterparts (*20*–*23*, *48*) (Fig. 2B and fig. S2, A to D), likely due to different geometric and/or buffering properties of somatic and dendritic compartments. We therefore used peak amplitudes to examine the relationship of Ca^2+^ activity between soma-dendrite ROI pairs.

In each dendrite-soma ROI pair exhibiting at least five Ca^2+^ events during the imaging session, we examined the degree of correlation of event amplitudes. All dendrite-soma ROI pairs had positive amplitude correlations; however, the dendritic event amplitudes were not simply a scaled version of somatic amplitudes. Rather, while correlations were generally high in dendritic ROIs relatively close to the soma (i.e., within ∼100 μm), at larger dendritic distances, the correlation strength decreased (Fig. 2, C and F; Spearman *R* = –0.498, *P* < 0.001, *n* = 290 ROI pairs in 23 cells from 14 mice; see also fig. S3A for data per cell) so that the variance of dendritic event amplitudes became increasingly less explained by the variance of somatic event amplitudes (fig. S3B), an effect that could not be accounted for by less accurate measurement of distal dendritic Ca^2+^ signals (Fig. 2, D and E). We found no significant difference in soma-dendrite correlation between cells with spatially tuned and untuned somatic activity in the given session, although a trend for lower correlations in nontuned cells was noted (Fig. 2G) (*31*). The relative decorrelation of distal dendrites from the soma likely arises from a combination of various factors, including passive dendritic properties, spatial and temporal variability and regulation of AP backpropagation, the nonlinearity of Ca^2+^ signal reporting of bAPs (*49*–*56*), and measurement constraints (see fig. S3, C to F, and Materials and Methods). Nevertheless, this distance-dependent amplitude correlation pattern suggests that Ca^2+^ activity in proximal dendrites closely tracks the activity of the soma, whereas more distal apical dendrites can have a larger degree of independence and may, in part, reflect local initiation of regenerative spikes that can propagate to other dendrites and to the soma (see below and fig. S3, C to F).

Consistent with this possibility, the dendrite/soma AR of Ca^2+^ events varied in a large range yet showed modulation with distance. The most proximal apical dendrites (<50 μm from soma) had overall higher event amplitudes than that at the soma (again possibly due to different geometric and/or Ca^2+^ buffering properties). In contrast, in more distal apical dendrites, the majority of events had larger somatic than dendritic amplitude (*31*) (i.e., AR < 1; see also note on dendritic event amplitude measurement in Materials and Methods). However, a fraction of Ca^2+^ events had substantially larger amplitude in the dendrite than at the soma (Fig. 2H and fig. S3, G and H), suggesting occasional dendritic origin of event generation.

Not only the dendrite-soma AR was variable but the duration of somatic Ca^2+^ events [quantified by the area under the curve (AUC)] also displayed large heterogeneity: While many transients, especially the ones with small to intermediate amplitude decayed fast and had small AUC, a fraction of the large-amplitude, sometimes complex events had much longer duration and thus nonlinearly higher AUC values (fig. S4A; see examples in Fig. 2B and fig. S2B).

These results altogether indicate that apical dendritic Ca^2+^ transients in CA3PCs comprised a mixture of events that are (i) generated at variable locations, including signals propagating from the soma to dendrites and signals propagating from dendrites toward the soma, and (ii) generated by heterogeneous mechanisms producing different kinetics.

### Slow global Ca^2+^ plateaus contribute to spatial tuning but rarely induce new PF formation

**Fig. 3.**
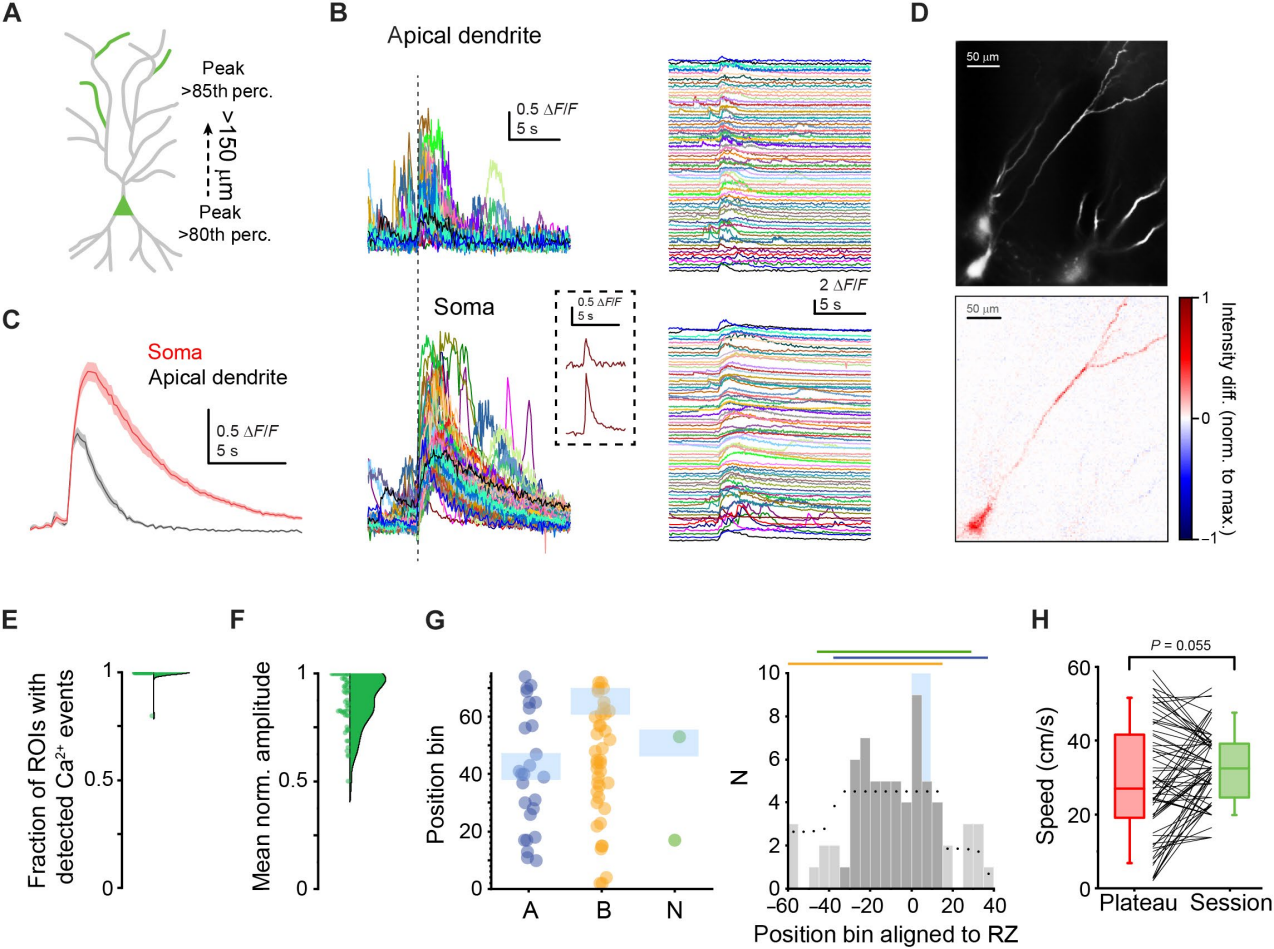
Identification and properties of putative Ca^2+^ plateaus. (**A**) Schematic of criteria to identify Ca^2+^ plateaus in the imaged ROIs (green). perc, percentile. (**B**) Somatic and dendritic Ca^2+^ transients of putative Ca^2+^ plateaus, aligned to their start. Inset shows one outlier Ca^2+^ transient (in soma and dendrite) that passed the amplitude criteria but had a fast time course. Traces are shown separately on the right. (**C**) Averaged binned somatic (red) and dendritic (black) waveforms constructed from Ca^2+^ plateaus without substantial subsequent contaminating activity during decay (*n* = 48 events). (**D**) Top: Mean 2P image of an example recording (cropped from the FOV). Bottom: Difference in fluorescence intensity during and before a Ca^2+^ plateau. The spatial footprint demonstrates strong global somatodendritic activity in the neuron. Same cell with ROIs and activity is shown in fig. S2Ab. norm., normalized; diff., difference. (**E**) Probability of detecting Ca^2+^ signal (with amplitude > 0.1 Δ*F*/*F*) among all ROIs belonging to a cell (soma and apical dendrites) corresponding to the Ca^2+^ events identified based on criteria in (A). (**F**) Mean normalized Ca^2+^ signal amplitude among all ROIs belonging to a cell (soma and apical dendrites) during the Ca^2+^ events identified based on criteria in (A). (**G**) Left: Location of Ca^2+^ plateaus in the familiar (A and B) and novel (N) VCs. Right: Distance of the spatial bin of Ca^2+^ plateau occurrence from the RZ start bin (events from all VCs pooled). RZ is indicated in blue. Expected frequency of the events occurring randomly is indicated by black symbols. VCs relative to RZ position are indicated on the top (colors as in left panel). Event frequency was not different from that expected from random localization (*P* = 0.572, chi-square test in the segment where VCs overlap; dark gray). (**H**) Running speed during the Ca^2+^ plateau compared to the median speed in the respective session.

We next sought to determine whether the different forms of Ca^2+^ transients described above might correspond to different forms of dendritic spikes, i.e., slow global Ca^2+^ plateaus and fast dendritic Ca^2+^ spikes described in CA3PCs in vitro. On the basis of our previous work (*10*, *40*, *42*), we expected that Ca^2+^ plateaus should be represented by Ca^2+^ transients with a large amplitude, slow time course, and global occurrence in the dendritic arbor. We first identified candidate events for Ca^2+^ plateaus based on their amplitude, i.e., events that had peak amplitudes in the upper 20% of the distribution of all pooled Ca^2+^ events at the soma and upper 15% of all pooled dendritic Ca^2+^ events in at least one apical dendrite more distal than 150 μm from the soma (*n* = 68 events in 17 cells; Fig. 3, A to C, and fig. S3, I and J). As expected, the selected events were typically detected throughout the whole dendritic arbor captured in the imaging plane (Fig. 3, D and E, and fig. S5), were among the largest Ca^2+^ transients within each ROI (Fig. 3F), and appeared also in basal dendrites (fig. S4, C and D). Inspecting the kinetics of the events selected by the above amplitude-based criteria revealed that they typically had a slow time course (Fig. 3, B and C). The averaged waveform (constructed from isolated Ca^2+^ events, i.e., that had no substantial contaminating activity on their decay phase) had a halfwidth of ∼5.6 s at the soma and ∼2.4 s at the dendrite (Fig. 3C). Accordingly, the somatic integrals of the selected events were among the largest values (fig. S4B). The large amplitude, prolonged time course, and global dendritic nature of the identified signals match the characteristics of slow plateau-like Ca^2+^ spikes previously described in these neurons (*10*, *40*, *42*) and putative Ca^2+^ plateaus in CA1PCs (*12*, *57*); thus, we will refer to these identified events as Ca^2+^ plateaus. Ca^2+^ plateaus occurred at various spatial positions in the two VCs (observed distribution not significantly different from randomly expected; *P* = 0.572, chi-square test) although were frequently observed at the beginning of the RZs where mice usually spend more time (Fig. 3G). The running speed of the animals during the time of Ca^2+^ plateaus tended to be lower than the median speed in the given session (Fig. 3H; Wilcoxon test, *P* = 0.055). Identification of Ca^2+^ plateaus based on the above criteria was corroborated by a uniform manifold approximation and projection (UMAP) analysis (*58*) using somatic and dendritic amplitudes and kinetics of Ca^2+^ signals, where plateaus mapped onto a segregated cluster of events (fig. S6).

Increased dendritic Ca^2+^ concentration associated with plateau potentials has been proposed to play a pivotal role in the induction of new PF formations in CA1PCs and CA3PCs via BTSP (*14*, *34*, *36*, *37*, *43*, *59*). However, the above studies measured somatic voltage or Ca^2+^ signals to identify Ca^2+^ plateaus that only provide indirect information regarding the associated dendritic activity. Our approach, where identified somatic Ca^2+^ plateaus are accompanied by large global dendritic signals (that are expected to induce BTSP-like synaptic plasticity), offers a strong basis to investigate the efficacy of Ca^2+^ plateaus to induce changes in somatic activity and tuning.

**Fig. 4.**
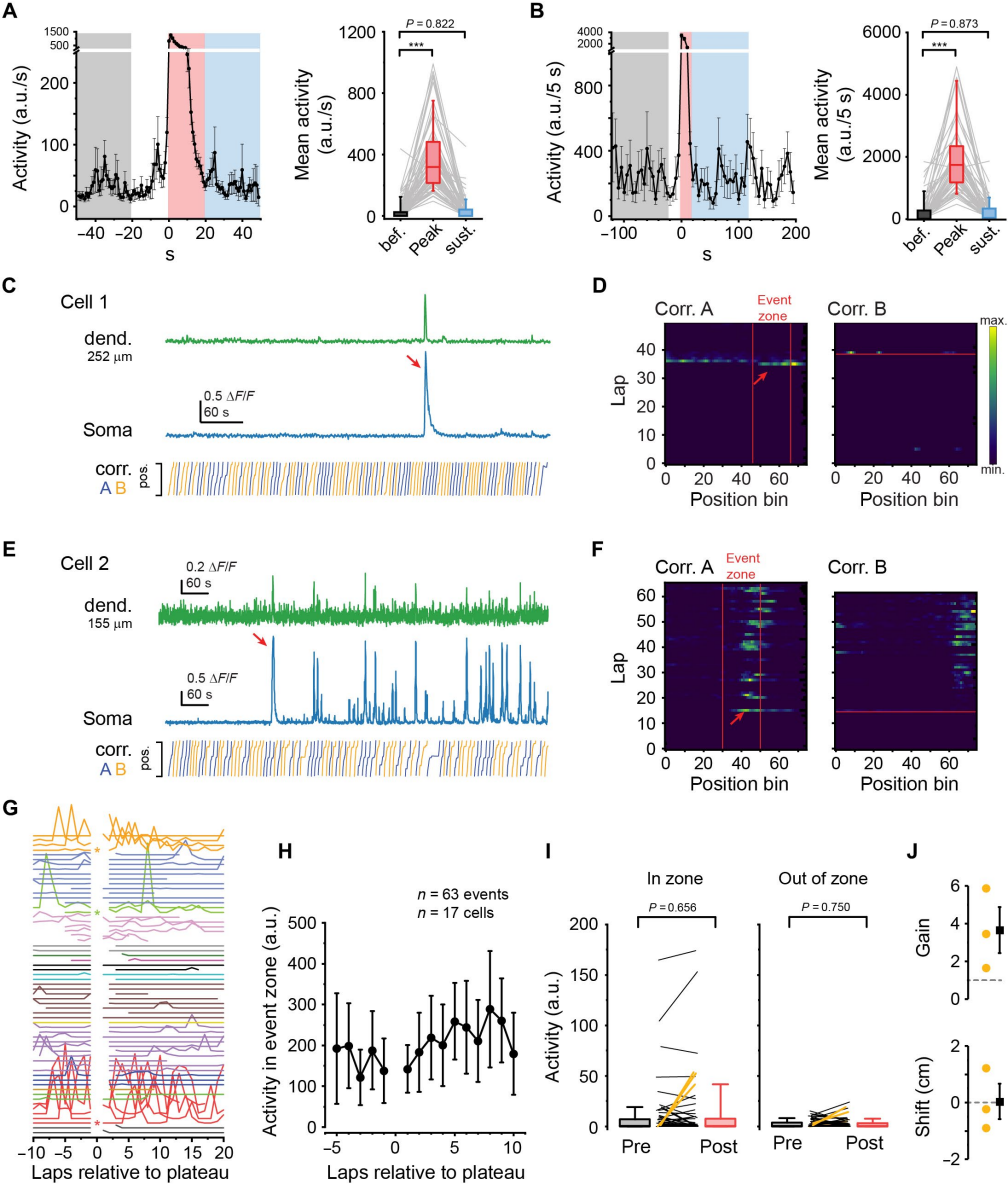
Ca^2+^ plateaus occasionally drive PF formation. (**A**) Left: Somatic activity (inferred spikes) before and after Ca^2+^ plateaus in 1-sec bins. Shadings indicate periods for calculating mean activity before (gray; bef.), within 20 s (red; peak), and ∼20 to 50 s after (blue; sust, sustained) the plateau time. Right: Somatic activity before and after *n* = 63 events. Friedman test (*P* < 0.001) with post hoc Wilcoxon tests (*P* < 0.001, *P* = 0.822). (**B**) Same as (A) but using wider time window and 5-s bins. *N* = 50 events. Friedman test (*P* < 0.001) with post hoc Wilcoxon tests (*P* < 0.001, *P* = 0.873). (**C**) Example cell with a Ca^2+^ plateau (red arrow) recorded at the soma (blue trace) and in an apical dendrite (green trace) that induced no change in somatic activity. Bottom: Animal position in the two VCs. dend., dendrite; pos., position. (**D**) Position-dependent lap-by-lap activity of the cell in (C) in the two VCs. Red arrow indicates Ca^2+^ plateau. Red vertical lines indicate the spatial zone where PF formation was investigated (“in-zone” activity). Red horizontal line in VC_B_ indicates the interleaved lap in VC_A_ where the plateau occurred. (**E** and **F**) Similar as (C) and (D) showing a cell where the Ca^2+^ plateau induced spatially tuned activity. (**G**) Each line represents summed in-zone somatic activity (inferred spikes) in laps before and after a Ca^2+^ plateau. Laps run during the plateau are blanked. Asterisks label plateaus that induced substantial increase of in-zone activity (see Materials and Methods). Lines are color coded by cells. (**H**) Lap-by-lap summary of the effect of Ca^2+^ plateaus on in-zone somatic activity (mean ± SEM). (**I**) Mean somatic activity/bin before (last three to five laps) and after (until lap 5 to 10) Ca^2+^ plateaus, calculated in-zone (left) and out-of-zone (right) in the corresponding VC. Lines: individual plateaus; orange: plateaus with substantially increased in-zone activity. *N* = 63 events; Wilcoxon tests. (**J**) Gain and shift of the PF for Ca^2+^ plateaus inducing spatially tuned activity.

First, we assessed the impact of Ca^2+^ plateaus on somatic activity in the temporal domain. To our surprise, comparison of the activity before and up to 2 min after the end of the plateau revealed neither instantaneous nor sustained difference (Fig. 4, A and B; see fig. S8, A and B, for data per mice). Next, we examined whether Ca^2+^ plateaus can elicit spatially selective increase in somatic activity, i.e., a new PF. Considering only those Ca^2+^ plateaus where the animal ran at least three laps before and five laps after the occurrence of the Ca^2+^ plateau in the respective VC (*n* = 63 events in 17 cells), we measured the mean somatic activity rate in the spatial zone where the plateau occurred (±10 spatial bins from event position within the given VC; Fig. 4, D and F; see Materials and Methods). We found that in 95% of the cases (*n* = 60 of 63 plateau events), somatic activity did not substantially change after the Ca^2+^ plateau (Fig. 4, C, D, G, and I; see Materials and Methods), and on average, no significant alteration in somatic activity was detected (Fig. 4, H and I, and fig. S8C for data per mice). However, in three cases, the Ca^2+^ plateau initiated a substantial increase in spatially tuned activity (i.e., a new PF was formed) lasting for at least 10 laps (*n* = 3 plateaus in three cells, 5%; in-zone/out-of-zone fold increase: 5.1 ± 1.4, range: 2.9 to 7.7; Fig. 4,E, F, G, and I, and fig. S4E). The three plateaus that effectively increased spatially tuned activity occurred in familiar VCs at variable speeds and spatial positions (fig. S4F), although the strongest effect was observed in a case where the plateau occurred in the RZ (Fig. 4F and fig. S4E). As expected from a BTSP-like PF induction mechanism, somatic activity in the PF after formation was weaker than the robust plateau-related activity during the formation lap (positive gain; see Fig. 4J and Materials and Methods) (*36*, *39*, *43*). The spatial position of the center of mass of the new PFs did not differ systematically from the position of the initiating Ca^2+^ plateau (no shift; see Fig. 4J and Materials and Methods). Although a small set, these data are in agreement with recent results suggesting that BTSP in CA3PCs exhibits a symmetrical temporal plasticity kernel (*43*), which is different from the asymmetrical rule in CA1PCs that leads to a backward shift in PF position after formation (*14*, *36*).

In addition to occasional PF induction by Ca^2+^ plateaus, several CA3PCs produced multiple, spatially clustered but temporally dispersed Ca^2+^ plateaus, which did not induce an immediate change in somatic activity in the first 10 subsequent laps and thus were not considered to induce BTSP, but their repeated occurrence at a similar spatial position produced statistically significant but unreliable spatial tuning of the neuron (fig. S4, G to I). Overall, 43% of all Ca^2+^ plateaus were observed in cells with spatially tuned activity in the given session, and 86% of these plateaus occurred in PFs. Altogether, these results demonstrate that Ca^2+^ plateaus contribute to the activity of CA3PCs in their PF (*43*) [similar to CA1PCs (*34*)] during navigation in virtual environments and in rare cases (∼5%) induce formation of a new PF, likely due to potentiation of spatially tuned afferent synaptic inputs from local recurrent collaterals (*43*).

### Fast dendritically initiated spikes are followed by transiently increased neuronal activity

**Fig. 5.**
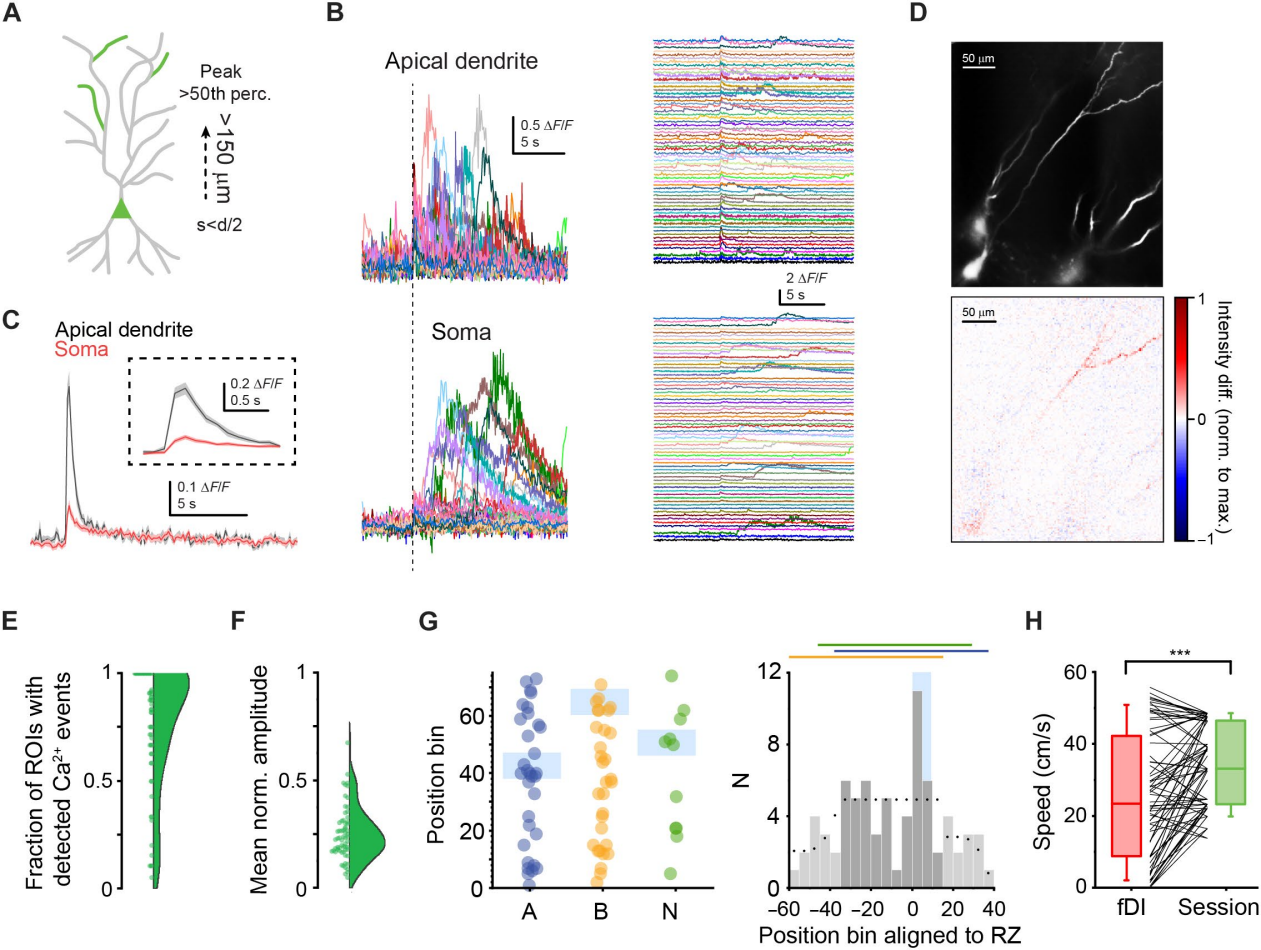
Putative locally generated Ca^2+^ spikes in distal apical dendrites of CA3PCs. (**A**) Schematic of criteria to identify fast dendritically initiated (fDI) spikes (see Materials and Methods). (**B**) Somatic and dendritic Ca^2+^ transients corresponding to fDI spikes identified as in (A), aligned to their start (*n* = 64 events from nine cells; 11 events where initial trunk was imaged instead of soma are not shown). Traces are shown separately on the right. Note that the events have fast time course but are often followed by subsequent, independent Ca^2+^ transients. (**C**) Averaged binned somatic (red) and dendritic (black) Ca^2+^ waveforms constructed from those fDI spikes that were not contaminated by subsequent activity during decay (*n* = 39 events). (**D**) Top: Mean 2P image of an example recording (cropped from the FOV). Bottom: Difference in fluorescence intensity during and before an fDI spike. The spatial footprint demonstrates strong distal dendritic and weak somatic change in fluorescence. Same cell as in Fig. 3D and fig. S2Ab. (**E**) Probability of detecting a Ca^2+^ event (with >0.1 Δ*F*/*F* amplitude) among all ROIs belonging to a cell during fDI spikes. (**F**) Mean normalized Ca^2+^ signal amplitude among all ROIs belonging to a cell during fDI spikes. (**G**) Left: Spatial distribution of fDI spikes in the familiar (A and B) and novel (N) VCs. Right: Distance of the spatial bin where the fDI spike occurred from the RZ start bin (events from different VCs pooled). RZ is indicated in blue. Expected frequency of the events occurring randomly is indicated by black symbols. VCs relative to RZ position are indicated on the top (colors as in left panel). Event frequency was not different from that expected from random localization (*P* = 0.120, chi-square test over the segment where VCs overlap; dark gray). (**H**) Running speed during fDI spikes compared to the median speed in the respective session.

We next focused our attention on locally generated regenerative activity in apical dendrites. To this end, we selected Ca^2+^ events whose amplitude in at least one distal apical dendritic ROI (≥150 μm from the soma) was relatively large (above the median of the distal dendritic amplitude distribution; fig. S3, I and J) and had at least twofold smaller, relatively modest amplitude at or near the soma (Fig. 5, A to C; *n* = 75 events in nine cells from seven mice; dendrite/soma ratio mean ± SEM: 4.81 ± 1.02, median: 2.93; see Materials and Methods). The selected events were often detected in only a part of the dendritic tree (Fig. 5, D and E, and fig. S5) and in about half of the cases at the soma as well (Fig. 5, B, D, and E), suggesting that they were caused by a voltage signal propagating over a relatively long distance (i.e., presumably dendritic spikes) rather than by highly localized dendritic activity (e.g., local synaptic inputs or nonpropagating NMDA spikes) and that they could often evoke AP at the soma. The spatial footprint of these events matched the dendritic morphology (Fig. 5D and fig. S5), ruling out that they were arising from cross-talk from GCaMP8s-labeled compartments of other neurons.

Ca^2+^ signals identified by the abovementioned amplitude-based criteria typically exhibited relatively fast kinetics (Fig. 5, B and C): The average binned waveform (constructed from *n* = 39 events that were not contaminated by subsequent Ca^2+^ transients) had a halfwidth of <0.72 s in the dendrite. The events, although could often be observed on other apical dendritic segments, exhibited heterogeneous amplitudes across ROIs (Fig. 5, E and F) and were typically not detected in basal dendrites (fig. S4, C and D). Although not directly comparable to voltage signals, the fast kinetics, propagating nature, and relatively large dendritic amplitude with weak or no somatic response altogether are consistent with the possibility that these events correspond to fast dendritic spikes mediated by Ca^2+^ (and possibly Na^+^) channels, which have been previously described in rat CA3PCs in vitro (*40*). We will refer to these events as fast dendritically initiated (fDI) Ca^2+^ spikes. Identification of fDI Ca^2+^ spikes based on the above criteria was corroborated by UMAP analysis, where they mapped onto a cluster of events (fig. S6A). fDI spikes were observed at various spatial locations in either familiar or novel VCs (observed distribution not significantly different from randomly expected; *P* = 0.120, chi-square test) although frequently occurred at the beginning of the RZs (Fig. 5G) and, accordingly, at relatively lower running speed (Fig. 5H; Wilcoxon test, *P* < 0.001). The majority (87%) of fDI events were observed in cells without spatially tuned activity in the given session, and of those occurring in tuned cells (*n* = 10 events in two neurons), only 30% were located at the PF.

**Fig. 6.**
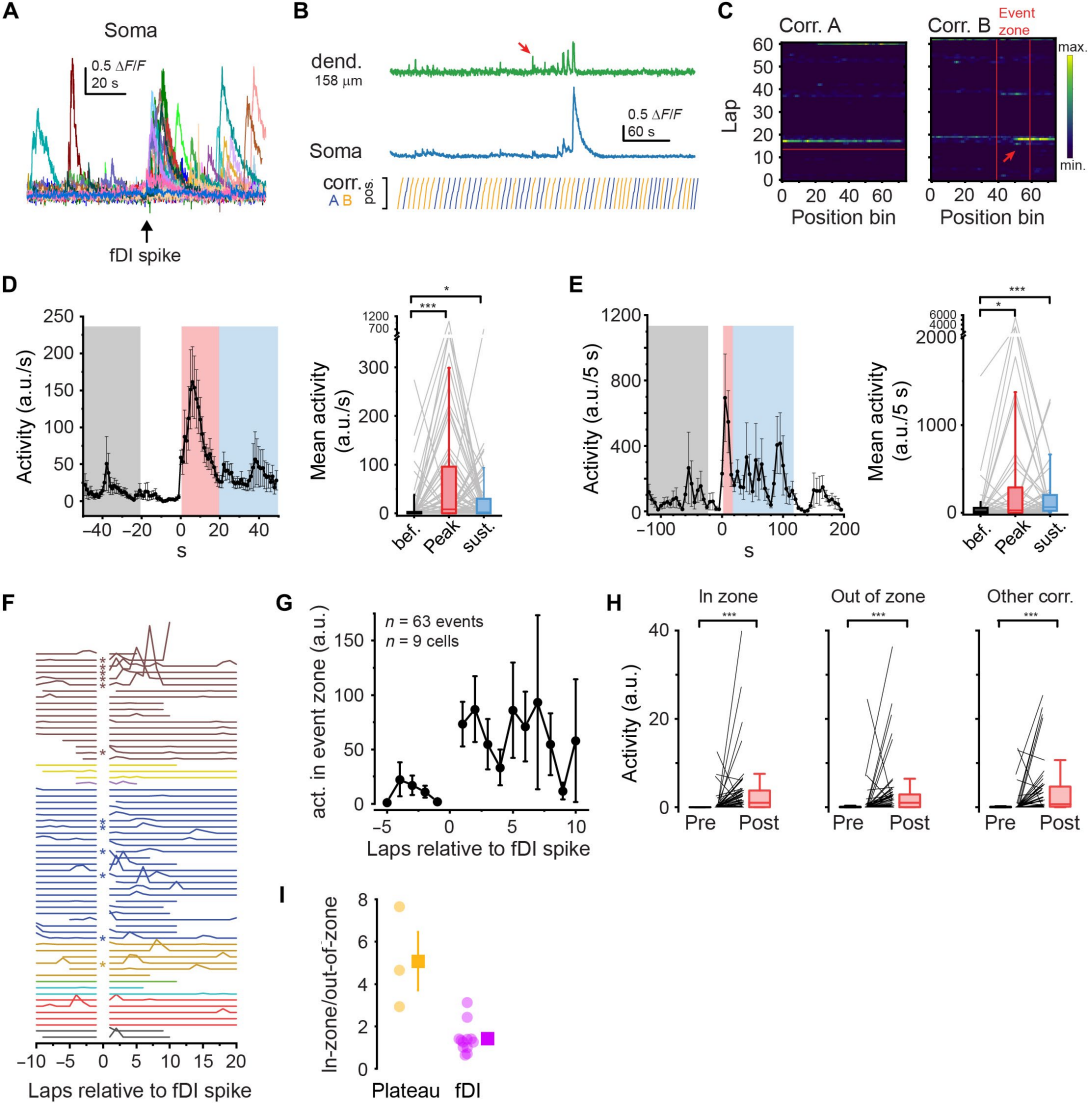
fDI Ca^2+^ spikes are associated with transient increase in neuronal activity. (**A**) Somatic Ca^2+^ activity ±50 s around fDI spikes. (**B**) Example recording segment with an fDI spike (red arrow) measured at the soma (blue) and in an apical dendrite (green). Bottom: Animal position in the two VCs. (**C**) Position-dependent lap-by-lap activity of the cell in (B) in the two VCs. Red arrow points to the fDI spike (with weak somatic signal). Red vertical lines in VC_B_ indicate the spatial zone of the event (for in-zone activity). Red horizontal line in VC_A_ indicates the interleaved lap in VC_B_ where the fDI spike occurred. (**D**) Left: Somatic activity (inferred spikes) before and after fDI spikes in 1-s bins. Shadings indicate periods for calculating mean activity before (gray), within 20 s (red; peak), and ∼20 to 50 s after (blue; sustained) the fDI spike (event bin excluded). Right: Summarized somatic activity change; *n* = 66 events, Friedman test (*P* = 0.001) with post hoc Wilcoxon tests (*P* < 0.001, *P* = 0.012). (**E**) Same as (D) but using wider time window and 5-s time bins (*n* = 44 events). Friedman test (*P* = 0.009) with post hoc Wilcoxon tests (*P* = 0.034, *P* < 0.001). (**F**) Total in-zone somatic activity in laps before and after individual fDI spikes (see Fig. 4G). Asterisks label substantial increase of in-zone activity. (**G**) Lap-by-lap summary of the effect of fDI spikes on summed in-zone somatic activity (mean ± SEM). act, activity. (**H**) Mean somatic activity/bin before (last three to five laps) and after (until lap 5 to 10) fDI spikes, calculated in-zone (left) and out-of-zone in the same VC (middle) and in the other VC (right). Lines: individual fDI spikes. *N* = 63 events; Wilcoxon tests (all *P* < 0.001). (**I**) Ratio of the in-zone and out-of-zone activity change (in the VC where the event occurred) for Ca^2+^ plateau–induced activity increase (*n* = 3; see Fig. 4, I and J) and fDI spike–induced activity increase (*n* = 12).

We next asked whether fDI Ca^2+^ spikes can be associated with any change in the somatic activity or tuning. Unexpectedly, we found that fDI spikes, even though they produced only small or undetectable somatic Ca^2+^ signals, were often followed by an increased rate of somatodendritic Ca^2+^ events for several seconds, especially of large slow Ca^2+^ events. Elevated activity was highest in the first ∼20 s but could be observed at lower intensity for ∼2 min after the fDI spike (Fig. 6, A to E; see fig. S8, D and E, for data per mice). This increased activity was, however, not specifically spatially tuned: When we examined the cases where the animal ran at least three laps before and five laps after the occurrence of the fDI spike in the respective VC, we found that the activity increased both within and outside of the spatial zone around the spike and even in the other VC (Fig. 6, F to H; *n* = 63 events). In cases with substantial in-zone increase (*n* = 12 events in 3 cells, 19% of all fDI spikes; Fig. 6F; see criteria in Materials and Methods), the in-zone and out-of-zone activity change was not different (*P* = 0.084, Wilcoxon test; the ratio of in-zone and out-of-zone activity change for fDI spikes versus plateaus: *P* = 0.017, Mann-Whitney test; Fig. 6I). The effect of fDI spikes on somatic activity did not depend on their spatial distance from the RZ (fig. S7A). Increased activity frequently followed even those fDI spikes that had very small amplitudes at the soma (fig. S7, B and C), and when we analyzed only those fDI spikes that could not be detected as events at the soma, the change in somatic activity was qualitatively similar to that measured with all fDI spikes included (fig. S7, D to G). Consecutive fDI events within a cell were not spatially clustered (fig. S7H). Altogether these results suggest that fDI spikes often precede (or evoke) a general increase in the activity of the neuron rather than induce plasticity of specific synaptic inputs.

## DISCUSSION

Here we measured Ca^2+^ activity in the apical dendrites and soma of individual CA3PCs expressing GCaMP8s to investigate the coupling of soma-dendrite activity and the types and effects of apical dendritic spikes. Although dendritic activity was generally well correlated with that of the soma, the strength of correlation decreased with distance. We identified two types of somatodendritic Ca^2+^ activities that could be explained by regenerative dendritic spikes: slow, large, global Ca^2+^ events as putative Ca^2+^ plateaus; and fast-decaying Ca^2+^ events with high dendrite-to-soma AR and limited propagation, representing putative dendritically initiated fast Ca^2+^ spikes. We found that Ca^2+^ plateaus contributed to spatially tuned activity of CA3PCs and on rare occasions could induce new PFs, via a mechanism consistent with proposed CA3 BTSP rules (*43*). In contrast, fDI Ca^2+^ spikes were often followed by a transient increase in somatic activity without spatial tuning.

Our work has four main findings. First, measuring concurrently somatic and apical dendritic activity in CA3PCs in vivo for the first time, we demonstrated substantial but distance-dependent coactivity of soma and apical dendrites: Branches in the first ∼100 to 150 μm were highly coupled with the soma, whereas more distal dendrites showed a wide range of correlations in event amplitudes, indicating larger independence. However, highly local activity (confined to a single short branch segment) was rarely detected in our experiments. With respect to soma-dendrite coupling, CA3PCs are therefore similar to other PC types including CA1PCs and layer 2/3 and layer 5 PCs in various cortical areas in that most Ca^2+^ events are detected in a wide extent of the dendritic tree, although differences in compartmentalization have been reported (*26*, *27*). The early branching structure of the apical dendritic arbor of CA3PCs could favor relatively independent function of dendritic subtrees—addressing this question will require measurements in larger areas of the apical dendritic tree with 3D imaging techniques. In addition, CA3PCs in vitro have NMDA receptor–mediated local dendritic spikes (*5*, *60*); however, we have not detected many localized Ca^2+^ events in apical dendrites. It is possible that local NMDA spikes are more prevalent in basal dendrites, which we have not examined in this study in detail.

Second, our results support the in vivo occurrence and functional relevance of two distinct types of apical dendritic Ca^2+^ spikes, whose characteristics are similar in many aspects to those previously found in CA3PCs in acute slices by our laboratory (*40*). Using patch-clamp recordings, we have revealed a variety of Ca^2+^ spike mechanisms in individual dendrites of rat CA3PCs, which were classified into two major groups: (a) slow (>50 ms) Ca^2+^ spikes typically generating after depolarizations following APs and (b) fast (∼5 to 10 ms) hybrid Ca^2+^/Na^+^ spikes generated locally in a stimulated dendrite and evoking only single somatic APs. In vitro Ca^2+^ imaging in different dendritic subtrees showed that slow Ca^2+^ spikes propagate efficiently throughout the whole dendritic arbor, whereas fast Ca^2+^ spikes remain relatively compartmentalized in the apical dendritic subtree where they were evoked, although the APs they trigger at the soma backpropagate to other dendrites inducing smaller fast Ca^2+^ signals (*40*). Consistent with these in vitro results, we have now found two types of specific Ca^2+^ events in CA3PCs in behaving mice using Ca^2+^ imaging in vivo. One is a global type with large amplitude both in soma and dendrites, typically lasting for several seconds [contrary to simple small-amplitude fast events whose decay matches the reported time course of GCaMP8s (*44*)]. Similar prolonged somatic Ca^2+^ events have been noted by a previous study in CA3PCs and were attributed to long CSBs (*61*). The characteristics of these Ca^2+^ events are also similar to putative Ca^2+^ plateaus associated with PF formation during virtual navigation in CA1PCs (*57*) and those accompanying CSBs in CA1PCs of anesthetized mice measured with combined electrophysiological and Ca^2+^ imaging methods (*12*). The other Ca^2+^ spike with fast kinetics (termed fDI Ca^2+^ spike) appears to be initiated in the dendrite based on its relatively large dendritic amplitude; however, it does propagate substantially as measured across several dendritic segments, indicating the involvement of voltage-gated channels, and it is often associated with a small coincident Ca^2+^ event in the soma or proximal dendrites, suggesting AP generation and backpropagation. Thus, these events cannot be generally defined as highly localized dendritic activity, yet they are also not completely and uniformly global (*7*). While it is not possible to distinguish between the sources of such regenerative fast Ca^2+^ signals (i.e., forward or backward propagating spikes) with our technique, in general, this activity is consistent with the unique fast Ca^2+^ spike mechanism previously described (*40*, *62*).

Third, we estimated the efficacy of global Ca^2+^ plateaus to induce new PF formation in CA3PCs. We took advantage of multidendritic Ca^2+^ imaging together with the soma and identified plateaus as those events that had large amplitude Ca^2+^ signals even in distal apical dendrites. These events typically produced prolonged large amplitude Ca^2+^ signals in all imaged dendritic segments [i.e., high branch spike prevalence (BSP) (*33*)], consistent with their expected ability to act as a robust general plasticity signal for BTSP. In CA1PCs, high BSP in perisomatic dendrites has been previously associated with PF precision, persistence, and formation (*30*, *33*), whereas tuft dendrites are variably recruited during PF formation (*57*). We confirmed that Ca^2+^ plateaus can initiate new PFs in CA3PCs (*43*); however, we found the probability of PF induction by plateaus to be low. Previous studies describing BTSP-like PF induction by prolonged CSBs mostly relied on somatic signatures of dendritic Ca^2+^ spikes, i.e., sustained depolarization with diminished AP amplitude [using intracellular voltage recordings (*14*, *34*, *38*, *63*) or voltage imaging (*59*)] or strong somatic Ca^2+^ signal (*35*, *36*), but dendritic activity has rarely been measured directly [but see (*33*, *57*)]. In addition, whether all similar events produced a new PF has not been reported. Thus, the probability of PF formation by spontaneous CSBs and the factors that determine this probability remained elusive. Notably, investigating PF formation in CA1PCs with somatic Ca^2+^ imaging during navigation in a long VC, we have recently found that many somatic Ca^2+^ events, which are at least as large and prolonged as a PF-inducing other Ca^2+^ event in the same cell, have no effect on neuronal activity and tuning (*39*). Our current analysis in CA3PCs corroborates this result: We found the probability of new PF formation by Ca^2+^ plateaus to be only ∼5%. However, we need to remain cautious about this result and keep in mind possible limitations in our experiments and analysis. One concern can be that, even with the high-sensitivity sensor GCaMP8s, we could have missed newly induced PF activity with sparse single-AP firing. While we cannot exclude this possibility, typical BTSP-induced new PFs in previous studies exhibit robust in-field activity [see examples in (*34*, *43*, *59*)], making this possibility unlikely. As another possible concern, virtual navigation may represent suboptimal conditions for PF formation (*64*) by providing less rich input to hippocampal PCs and thus lowering the likelihood of somatic firing even by potentiated synapses, a possibility we cannot exclude either. Last, the impact of GCaMP8s on Ca^2+^ homeostasis (a critical factor for many synaptic plasticity processes) may interfere with the signaling pathway(s) by which Ca^2+^ plateaus induce BTSP; therefore, experiments with complementary technical approaches will be important to confirm our results and assess the contribution of different cellular mechanisms to PF formation in CA3PCs. Nevertheless, the current data suggest that the induction of new PFs by Ca^2+^ plateaus in CA3PCs may require an additional gating factor [e.g., increased neuromodulatory tone (*42*)].

As the fourth main result, we found that fDI Ca^2+^ spikes are often followed by increased rate of neuronal activity—in particular, by subsequent slow Ca^2+^ events—for tens of seconds after the end of the spike. The temporarily elevated activity was, however, not spatially tuned. While the mechanistic explanation of this phenomenon is currently unclear, we contemplate several possibilities. One potential scenario is that the initial occurrence of the fDI spike coincides with (and perhaps is caused by) the start of increased activity of afferent synaptic inputs, which may arrive from the entorhinal cortex, recurrent collaterals, mossy fibers, or a combination of these sources. A second possibility is that the occurrence of the fDI spike induces an increase in dendritic excitability (e.g., via modulation of dendritic ion channel function), which leads to enhanced neuronal response to unchanged synaptic inputs along the VC track. It is notable that Ca^2+^ plateaus, which are much more robust events than fDI spikes, produced no similar transient activity increase, which implies (i) that the effect is not simply due to elevated [Ca^2+^] and (ii) that the two dendritic spike types may initiate fundamentally different plasticity mechanisms: synaptic versus intrinsic. Both of the above processes (increased input or plasticity of excitability) may be facilitated by phasic neuromodulatory effects, for example, by the cholinergic or noradrenergic systems. Future experiments measuring synaptic activity and neuromodulatory signals along with dendritic Ca^2+^ or voltage (*65*) may shed light on the underlying mechanisms and help to dissect the contribution of the different factors.

In conclusion, our results uncover multiplexed organization of dendritic function in CA3PCs: Superimposed on a generally close dendritic coupling to somatic activity, the dendritic tree is endowed with the ability to produce multiple dendritic spike types with different properties and functional roles that may enrich the flexibility of CA3PCs to generate environment-specific spatial representations.

## MATERIALS AND METHODS

### Animals

Experiments were conducted in accordance with the guidelines of the European Communities Council Directive (86/609 EEC), the Hungarian National Scientific Ethical Committee on Animal Experimentation regulation no. AA2.0/2015, and the Animal Care and Use Committee of the Institute of Experimental Medicine and with the approval of the Committee for Scientific Ethics of Animal Research of the Government Office of Pest County Department of Food Chain Safety, Veterinary Office, Plant Protection and Soil Conservation Budapest under the project number PE/EA/676-7/2021. All efforts were made to minimize pain and suffering and to reduce the number of animals used. Male wild-type FVB/AntJ (for AAV-mediated GCaMP8s expression) or heterozygous C57BL/6J-Tg(Thy1-GCaMP6s)GP4.3Dkim/J mice (*66*) (in a subset of experiments; fig. S1, E and F) were used. Animals were housed in a vivarium in groups (two to five mice per cage) on a reversed 12-hour light/dark cycle and moved to a ventilated animal cabinet after their first surgery. Experiments were performed during the dark phase of the cycle.

### Surgery

At the age of 33 to 43 days, mice were anesthetized with ketamine-xylazine (ketamine: 100 mg/kg; xylazine: 10 mg/kg; both from Produlab Pharma BV, Netherlands), head fixed in a stereotaxic frame (Kopf Instruments), and injected with a combination of low titer Cre virus [AAV9-CMVs-Cre (#105537-AAV9); batch titer: 10^13^ genome copies per milliliter (GC/ml), diluted to 1:4000] and a high titer flexed GCaMP8s virus [pGP-AAV9-syn-FLEX-jGCaMP8s-WPRE (#162377-AAV9); batch titer: 10^13^ GC/ml, diluted to 1:10; all viruses from Addgene] into two sites in the CA3 area of the left hippocampus through a small craniotomy (1: AP: −1.3 mm, ML: −1.5 mm, and DV: −1.85 mm from bregma; and 2: AP: −1.3 mm, ML: −1.7 mm, and DV: −1.9 mm, 70 to 90 nl per injection site) using a glass pipette mounted on an injector (World Precision Instruments). In a subset of experiments, we used a lower dilution of Cre injected at two or three sites (1:400 diluted; coordinates: 1: AP: −1.3, ML: −1.5, DV: −1.85; 2: AP: −1.3, ML: −1.7, DV −1.9; 3: AP: −1.3, ML: −1.7, DV: −1.95, 100 nl in each site) to increase the density of CA3PC labeling for population imaging. Virus dilutions were made in phosphate-buffered saline. During all surgeries, body temperature was maintained at physiological levels with a heating pad, and the eyes were protected using an eye cream (Opticorn A, Werfft-Pharma Kft., Hungary). After surgery, the scalp was sutured, and the animal was monitored during recovery from anesthesia before returning to the home cage.

Typically 10 days (range: 3 to 20 days) after recovery from virus injection, mice were surgically implanted with a hippocampal cranial imaging window and headpost, according to previously established protocols (*67*, *68*). Briefly, under isoflurane (1.5 to 2.5%) anesthesia and with the application of local anesthetic (lidocaine; 0.05 ml; Hungaropharm, Hungary) subcutaneously (sc), a 3-mm-diameter craniotomy was made in the exposed and cleaned skull over the left dorsal hippocampus. After gentle removal of the dura, the underlying cortex was slowly aspirated with continuous irrigation with chilled Ringer solution until the external capsule was exposed. Top-most layers of the external capsule were lightly peeled away. A custom-made imaging cannula (3-mm diameter × 1.6-mm height) made by adhering (Norland optical adhesive #81) a 3-mm round glass cover slip (Deckglaser) window to a stainless steel cylinder was inserted into the craniotomy. The imaging cannula was cemented to the skull with black dental acrylic (Unifast Trad with carbon powder added to the cement) along with a light but durable custom-designed titanium alloy (TiAl6V4) headpost for head fixation. Buprenorphine (0.1 mg/kg, sc; Bupredine, Le Vet. Beheer B.V., Netherlands) was administered after the surgery to minimize postoperative discomfort. After monitoring recovery from anesthesia, mice were returned to the home cage and recovered for 4 to 7 days with free access to water. Carprosol (50 mg/ml; Rycarfa, Krka, Slovenia) was supplemented in the drinking water for the following 3 days of postoperative care.

### Behavioral training

A minimum of 4 days after the implant surgery, mice were introduced to a water restriction protocol (keeping their weight above 80% of their predeprivation body weight) and hand habituation. On the first day of training, mice were placed in a translucent box onto the treadmill in the experimental apparatus and were allowed to lick water from the lick port. On the following day, mice were head fixed in the setup and began the training to run on a cueless treadmill belt by rewarding them with water drops of variable preset size and adjusting the distance between water delivery sites (“lick and run training”). During this training phase the, virtual reality (VR) monitor screens were on, displaying a corridor with simple gray wall but no distinct visual cues other than the corridor end wall. After several (usually 3 to 4) days, when mice voluntary ran more than 30 laps, they were introduced to the goal-oriented spatial discrimination task, in which they had to navigate in (and discriminate between) two 160-cm-long, pseudorandomly presented virtual linear corridors with distinctive wall patterns, and lick selectively in a 18-cm-long RZ that was located at different locations (starting at 82 and 129 cm in VC_A_ and VC_B_, respectively) along the two corridors but had the same wall pattern (Fig. 1B). Mice had to lick operantly in the RZ to trigger the delivery of one drop of water reward. When the mice reached the end of a VC, they were teleported to the start of the next lap. The two corridors were presented in a pseudorandom order to obtain comparable numbers of trials for analysis from both. Learning and performance were assessed on the basis of achieving a high rate of reward collection and exhibiting corridor-selective anticipatory behavior (slowing down and licking before the RZ). In a subset of animals with high-performance behavior, we switched during the imaging session to a novel corridor of the same length but with a distinct wall pattern and RZ with texture and location different from those in the familiar corridors (starting at 98 cm). In case the performance of the animal was poor at the beginning of a session, water was provided nonoperantly in the RZ for a few laps (as a reminder of the task) before returning to operant behavior. Nonoperant water delivery was also used in the novel VC if the animal’s behavior did not improve over many laps. Behavioral training typically lasted for ∼2 weeks. Two-photon (2P) imaging sessions (duration: 10 to 26 min, often embedded in longer behavioral sessions without imaging) were performed at various stages of learning, within 19 to 51 days after virus injection (typically within 5 weeks). Mice usually performed one or two sessions a day, with no training and imaging during weekends.

### Behavioral apparatus and virtual reality

The behavioral setup (Luigs & Neumann; Fig. 1A) consisted of a treadmill covered with a cueless black fabric belt (94 cm long), surrounded by three monitors at 90^o^ angles with each other projecting linear virtual environments. A lick port (a water spout combined with a lick sensor) was used for lick detection and water dispersion. Locomotion (speed) was recorded by tracking the rotation of the treadmill wheel using an optical rotary encoder. For synchronization of behavioral recordings and image acquisition, transistor-transistor logic (TTL) pulses of increasing durations were generated on the behavioral computer and were recorded by PrairieView (Bruker) on the imaging computer for offline pairing. Linear virtual environments were constructed using a modified version of the script written by Luigs & Neumann in PyOgre. Images for the textured corridor walls were generated in Adobe Photoshop. The behavioral apparatus was controlled using the LabVIEW graphical programming environment.

### Evaluation of behavioral performance

We quantified behavioral performance using a scoring system consisting of the following components: correct lap proportions, anticipatory lick and speed indices (within a VC), and lick and speed selectivities (across VCs). The correct lap proportion (*P*_correct_) for a given VC is the proportion of laps in which the animal licked within the RZ. To calculate lick and speed indices, we defined a prereward zone (preRZ; five spatial bins immediately preceding the beginning of the RZ) and a control zone (CZ; five spatial bins preceding the beginning of the RZ by 30 cm) where reward should not be expected by the animal. The lick index is defined as the selectivity of the average lick rate ($\hat{l}$) between the preRZ and the CZ within a given VC

$$l_{\text{idx}}=\frac{{\hat{l}}_{\text{preRZ}}-{\hat{l}}_{\text{CZ}}}{{\hat{l}}_{\text{preRZ}}+{\hat{l}}_{\text{CZ}}}$$

The speed index within a given VC is calculated similarly, as the selectivity of the average speed ($\hat{v}$) between the preRZ and the CZ

$$v_{\text{idx}}=\frac{{\hat{v}}_{\text{preRZ}}-{\hat{v}}_{\text{CZ}}}{{\hat{v}}_{\text{preRZ}}+{\hat{v}}_{\text{CZ}}}$$

Speed and lick selectivities across the VCs were calculated the following way. We identified the preRZ of VC_A_ as described above, which we call the selectivity zone (SZ). Then we calculate the speed (or lick) selectivity by calculating the selectivity of the average speed (or lick rate) across the SZ in both familiar VCs

$$l_{\text{sel}}=\frac{{\hat{l}}_{A,\text{SZ}}-{\hat{l}}_{B,\text{SZ}}}{{\hat{l}}_{A,\text{SZ}}+{\hat{l}}_{B,\text{SZ}}}$$

$$v_{\text{sel}}=\frac{{\hat{v}}_{A,\text{SZ}}-{\hat{v}}_{B,\text{SZ}}}{{\hat{v}}_{A,\text{SZ}}+{\hat{v}}_{B,\text{SZ}}}$$

Last, we quantify the overall performance of an animal in one session by calculating the sum of these components, termed behavior score (BS)

$$\begin{array}{c} \text{BS}=P_{\text{correct},A}+P_{\text{correct},B}+l_{\text{idx},A}+l_{\text{idx},B}+\\ l_{\text{sel}}+c\cdot \left(v_{\text{idx},A}+v_{\text{idx},B}+v_{\text{sel}}\right) \end{array}$$

The constant denoted by c is meant to normalize speed indices and speed selectivity into the range of 0 and 1. All other components of the behavior score fell in this range; therefore, to avoid penalizing the contribution of speed indices and selectivity in the summation of the BS components, we normalized their values accordingly. Because the typical values of speed indices and selectivity fell between 0 and −0.45, we defined *c = −2.2* to achieve this normalization. We note that with speed indices and/or selectivities lower than −0.45 the value may become larger than 1 after normalization, but this rarely happened and did not influence BS substantially.

### In vivo 2P imaging

Calcium imaging in head-fixed, behaving mice was performed as previously described (*68*) using a 2P microscope (Bruker Investigator) equipped with an 8-kHz resonant scanner and a Ti:Sapphire laser (Chameleon Vision II, Coherent) tuned to 920 nm. For image acquisition, we used a Nikon 16x water-immersion objective [0.8 numerical aperture (NA), 3 mm working distance], and fluorescent signals were collected by a GaAsP photomultiplier (PMT) tube (Hamamatsu). Capturing stray light from the virtual reality monitor by the PMT was blocked using a custom-made, 3D-printed black plastic cone that fitted the outer rim of the head plate, and a thick black balloon was attached to the cone and the lens. To adjust the angle of the imaging window to the plane of the front lens of the objective, we set the angle of the head-fixed mouse’s head with two goniometers (Edmond Optics). Image series (512 × 512 pixels, at 1.3 to 3.2× zoom, ∼260 to 650-μm side) were acquired using the PrairieView software, which also recorded TTL pulses with variable durations, generated at the beginning of each lap, for offline alignment of imaging and behavior data. Imaging sessions with only few laps run, bad imaging quality, or severe motion were discarded. Data analyzed here were obtained in 76 sessions from 17 mice (duration: 10 to 26 min, spread over several days, one or two sessions per day), either in a single plane at ∼30 Hz (*n* = 38 sessions) or in two planes 30 to 80 μm apart using a piezo objective positioner at ≥5.97 Hz (mean: ∼9.62 Hz; *n* = 38 sessions). After time-series imaging, z-stacks of the FOV were acquired at 2-μm steps. FOVs shown in the figures are mean images of time series.

### Analysis of neural Ca^2+^ activity

#### Preprocessing of GCaMP8s-mediated Ca^2+^ data

Motion correction of the imaging data was performed using Suite2p (version 0.10.3) (*69*). The resulting motion-corrected image series were analyzed using custom-written scripts in Python. We first created a mean image of the time series, where the fluorescence intensity was log scaled for better visualization aiding ROI segmentation. For analysis of somatic activity, ROIs were drawn around all somata visible in the FOV. For analysis of somatodendritic activity of an individual neuron, ROIs were drawn manually around the soma and around each dendritic segment that had at least ∼5- to 20-μm length located in the focal plane, carefully following the outline of the compartment. Longer dendritic segments were divided into multiple neighboring ROIs of ∼15- to 20-μm length. In the case of sparse labeling, signal contamination from other cells was negligible. Identification of dendritic segments belonging to a given soma was based on tracing them offline on the z-series and was further aided by their often coincident activity with the soma, as well as by constructing a pixel correlation map that computed the correlation of the fluorescence of each pixel in the FOV with the selected ROI (fig. S2C). The video used for calculating this pixel-wise correlation was smoothed by convolution with smoothing kernels

$$\left[\begin{array}{ccc} 0.1 & 0.1 & 0.1\\ 0.1 & 0.2 & 0.1\\ 0.1 & 0.1 & 0.1 \end{array}\right]$$

and

[0.25 0.5 0.25]

in the spatial and time domains, respectively.

Average fluorescent signals from the pixels belonging to each ROI were measured (without background subtraction), and the relative fluorescence change was calculated as Δ*F*/*F* = (*F* – *F*_0_)/*F*_0_, where *F*_0_ is the mode of the binned raw fluorescent signal *F* (number of bins = 100). We then baseline corrected these traces by subtracting the baseline drift, which we obtained by applying a minimum then a maximum filter with a 60-s-long moving kernel to the Gaussian smoothed (SD: 10 frames) raw Δ*F*/*F* traces, similar to Suite2p (*69*). For all event-based analyses, we then applied Gaussian smoothing of the baseline-corrected Δ*F*/*F* signal with an SD of 100 ms (single-plane imaging) or 200 ms (dual-plane imaging).

The ROI-specific baseline noise SD was estimated from the baseline-corrected (but not smoothed) Δ*F*/*F* signals by calculating the SD in a 101-frame-wide sliding window and taking the 10th percentile of these values. While fluorescence was variable across cells in a given FOV, we found no significant correlation between resting fluorescence and Ca^2+^ event rate of neurons (measured in recordings with relatively dense labeling).

We note that when imaging small, dim structures deep in tissue, the relative contribution of background fluorescence and the small PMT offset to the measured signal is not negligible. These factors reduce the amplitude of Δ*F*/*F* signals, likely contributing to the observed decrease in dendritic event amplitudes with distance from the soma. Therefore, reported Δ*F*/*F* values, especially in distal dendrites, can be interpreted as a lower bound. More broadly, the direct comparison of somatic and dendritic Ca^2+^ signal amplitudes is confounded by discrepancies in compartment size, indicator concentration, signal kinetics (faster dendritic signals are more affected by undersampling and smoothing), and the relative impact of background and potential indicator saturation. Ca^2+^ traces of most of the example recordings were slightly smoothed for display purposes by averaging three data points within a moving window.

### Preprocessing of Ca^2+^ data from Thy1-GCaMP6s mice

After motion correction, segmentation of ROIs and extraction of raw GCaMP6s fluorescent Ca^2+^ signals were performed using Suite2p (*68*, *69*). ROIs of putative CA3PCs were manually curated to include ROIs falsely identified as “not cell” by the algorithm and to exclude occasional multiple cells coregistered as one ROI (based on shape and size) and ROIs with contaminated activity (based on overlap between neighboring ROI signals) in a conservative manner, using Suite2p graphical interface. To obtain an unbiased dataset (also including silent cells), ROIs on somata that were categorized as “no cell” by Suite2p were manually included in the “cell” pool. After extracting the raw fluorescence, subsequent data analysis was performed similar to that for the GCaMP8s-mediated data.

### Event detection and analysis

For identification of Ca^2+^ events, we used an event detection algorithm on the baseline-corrected and smoothed Δ*F*/*F* signals. Initial candidate events were identified as local maxima of the trace where the signal exceeded four times the noise SD (see Ca^2+^ data preprocessing) for the given ROI. In addition, to eliminate false positives due to occasional slow baseline drifts going above threshold, we took the minimum positive value of the preceding 10 frames before the detected event peak as the local baseline and accepted events if the signal exceeded two (when analyzing different ROIs of individual cells) or three (when analyzing somatic signals of a population of cells) times the noise SD. When multiple candidate events were detected on the suprathreshold part of the trace, we only kept the ones that were at least two or three times the noise SD higher than the preceding local minimum (thus eliminating mostly small signals on the decaying part of large slow-decaying transients). For somatic Ca^2+^ event rate measurements (Fig. 1), we did not perform further curation of transients. For the analysis of somatodendritic signals, the detected transients were inspected and manually curated to eliminate any remaining signals apparently arising from noise (mostly small events with spurious kinetics), as well as events occurring during the declining phase of large, slowly decaying Ca^2+^ signals, because their amplitude could not be similarly determined in the compartments with different decay kinetics (i.e., soma and dendrites). We note that smoothing results in underestimation of the peak amplitude of Ca^2+^ transients, especially of fast events. The Ca^2+^ signal amplitude (peak Δ*F*/*F*) corresponding to the identified events was measured in each of the imaged compartments (including those where the event itself could not be detected) within 0.4 s from the peak, creating an amplitude matrix for each event across all ROIs. For building this event amplitude matrix, we went through the ROIs in a predefined order starting with the soma as a seed and gradually moving toward the more distal ROIs. Starting with the soma for each detected event, we checked whether in the other ROIs there was also an event detected in the coincidence window (±0.4 s). We always considered the event with the highest peak as the corresponding event amplitude and ignored all other events within the coincidence window. In case there was no event detected in the coincidence window for a given ROI, the maximum value of the trace in the same coincidence window was used as corresponding amplitude. Then we proceeded onto the rest of the ROIs as seeds for event detection to find any events so far unaccounted for and repeated the same steps.

AUC analysis of somatic Ca^2+^ signals (fig. S4A) was performed on a subselected group of the events that were not contaminated by subsequent transients before returning to baseline. AUC of these events was also calculated using the baseline-corrected smoothed Δ*F*/*F* signals by summing up the area between zero and the calcium signal where the signal exceeded four times the noise SD. For the putative plateaus (some of the largest transients with often multiple peaks), we calculated AUC the same way, but occasionally the boundaries of the transient, which could be considered the same event, was manually defined (although note that these start and end points were always above the four times noise SD threshold).

### Comparison of activity between soma and dendrite

For each soma-dendrite ROI pair, the peak amplitude of every event observed in any of the two ROIs was plotted against each other, and the correlation coefficient (*r*) and adjusted coefficient of determination (*R*^2^) values of a linear fit were calculated. Next, the ratio of the dendritic and somatic amplitude (D/S) was calculated for each event (events with <0.1 Δ*F*/*F* amplitude in both the soma and the dendrite were excluded) in each ROI pair.

For the distribution of somatic amplitudes of Ca^2+^ transients, the dataset includes those cells whose soma was in plane and that had at least one dendritic ROI. Detected transients with <0.1 Δ*F*/*F* amplitude were excluded.

Distance of dendritic ROIs from the soma was measured using ImageJ by drawing a segmented line following the dendritic trajectory, aided by z-stacks of the cell and by pixel correlation maps (see fig. S2, C and D). In case of ambiguity of the dendritic path or incomplete z-stack, the shortest path between visible dendrite segments was drawn. Because of the almost horizontal orientation of CA3PCs, dendrite tracing was typically done in 2D without considering the *z* dimension; therefore, dendritic distances are considered as estimates.

### Definition and analysis of different types of putative dendritic spikes

Putative dendritic spikes were selected based on Ca^2+^ signals measured at the soma and in distal apical dendritic ROIs (defined as located ≥150 μm from the soma). The selection criteria were chosen arbitrarily and relatively conservatively to identify Ca^2+^ events with specific features consistent with expected properties of dendritic spikes.

Putative slow global Ca^2+^ spikes were identified as signals that (i) at the soma had peak amplitude larger than the 80th percentile of all somatic event amplitudes (≥1 Δ*F*/*F*) and (ii) in at least one distal dendritic ROI had peak amplitude larger than the 85th percentile of all dendritic event amplitudes (≥0.491 Δ*F*/*F*). The kinetics of Ca^2+^ plateaus were analyzed including only those events that were not contaminated with substantial subsequent activity within 20 s.

Putative local dendritically initiated spikes were identified as events detected in at least one distal apical dendritic ROI that had (i) a dendritic event amplitude larger than the median of the cumulative distribution of all events in all dendritic ROIs ≥ ∼150 μm from the soma (fig. S3J; ≥0.158 Δ*F*/*F*) and (ii) at least 2× larger amplitude than that at the soma (or the proximal trunk if the soma was out of focus; *n* = 11 events in one cell. These events were not included in the analyses in Figs. 3, E and F, and 5, E and F). We excluded events with >0.7 Δ*F*/*F* amplitude at the soma or in the initial stem of the trunk (<20 μm from the soma) and events that had both >0.5 Δ*F*/*F* amplitude in the initial stem of the trunk and >0.3 Δ*F*/*F* amplitude at the soma. We note that because our measurement is restricted to a small fraction of the dendritic tree, (i) we probably often image the dendrites relatively far from the spike generation site, and thus the propagating signal may remain subthreshold to our identification criteria; and (ii) somatic Ca^2+^ events without large dendritic Ca^2+^ signal may still be induced by fDI spikes that originate in branches that are not in the imaged areas.

The kinetics of the identified fDI events were analyzed only in cases where the Ca^2+^ measurement was done in the soma, and which were not contaminated by subsequent activity within ∼20 s. The events were aligned according to the imaging frame where the Δ*F*/*F* signal began to rise above baseline. For the halfwidth analysis, due to different frame rates in different imaging sessions, we computed the mean trace after we binned all data using the bin width defined by the lowest sampling rate. The halfwidth was defined as the time difference between the frames before and after the half maximum amplitude value. One fDI event was detected before the animal started the task and was included only in kinetic analysis.

To examine the spatial footprint of selected events in the dendrites (fig. S5), we first calculated the mean of three frames both during the event and during the resting period before the event. The two images were subtracted and spatially slightly smoothed (using the same 3 by 3 pixels smoothing kernel as described previously). The spatial footprint of the dendritic segment matched the dendritic morphology in the events selected as putative dendritic spikes and excluded contamination by other cellular structures overlapping with the dendrite.

### Uniform manifold approximation and projection

For every detected event for which we could reliably measure the somatic AUC (see event detection and analysis) and had at least one distal (at least 150 μm from the soma) dendritic ROI in the focal plane, we collected six parameters: (i) the somatic amplitude (soma ampl.), (ii) the somatic amplitude normalized to the largest somatic amplitude in the given recording session (soma ampl. norm.), (iii) the largest distal dendritic amplitude (dendr. ampl.), (iv) the largest distal dendritic amplitude normalized to the maximum amplitude in the corresponding ROI in the given recording session (dendr. ampl. norm.), (v) the natural logarithm of the largest distal dendritic amplitude divided by the somatic amplitude (dendr./soma), and (vi) the somatic AUC divided by the somatic amplitude (soma AUC/ampl.) that characterizes the kinetics of the event. After *z*-scoring the above parameters, we used the umap-learn package in Python to perform a UMAP analysis (*58*) to capture the underlying structure in the data in two dimensions. For the results shown here, we used the following settings: n_components = 2, init = ‘random’, random_state = 0, n_neighbors = 50, and min_dist = 0.05, but got qualitatively similar results with a wide range of n_neighbors and min_dist values. We used the AgglomerativeClustering hierarchical clustering method (with the Ward linkage criterion) available in the sklearn.cluster package in Python to identify clusters in this 2D projection of the data.

### Analysis of the impact of selected Ca^2+^ events on somatic activity

To determine the impact of selected Ca^2+^ events on the somatic activity, we calculated lap-by-lap binned inferred spike rate maps for the cells in both corridors similarly to that described in “Analysis of spatial tuning,” with the only difference being that the inferred spikes were removed below three times the noise SD (instead of four).

The time point and spatial position of occurrence of fDI spikes were defined at the imaging frame of their peak Ca^2+^ signal, whereas the time point and spatial position of occurrence of slow plateaus were defined at the imaging frame where the first large peak of the Ca^2+^ signal occurred (either at the soma or in dendrites). The speed of the animal during an event was calculated as the average speed in the spatial bin of the event.

The impact of dendritic spikes on spatially tuned somatic activity was determined including only those cases where the mouse ran at least three laps before and five laps after the occurrence of the Ca^2+^ event in the respective corridor. We focused on the activity in the spatial zone of the detected event, which we specify as the spatial bin corresponding to the time of the transient (as defined above) plus-minus 10 spatial bins (or until the beginning or end of the corridor) in the given VC, while also monitoring the activity outside the zone and in the other corridors. We calculated the activity as the mean of the position-binned inferred spike rates in the given spatial zones in each lap. Inferred spikes corresponding to the same event but in subsequent laps (on the decaying part before the Ca^2+^ signal returned to baseline) were removed in this analysis.

Because of large lap-to-lap fluctuation of activity and frequent silent laps, we considered an event to induce substantially increased activity in the spatial zone if the following criteria were met: After the event, (i) activity was detected in the event zone in at least 3 of the subsequent 10 laps, (ii) activity (mean inferred spike rate) increased by at least 2 a.u. and to at least 10-fold compared to the mean in the zone during the last three to five pre-event laps (in case of no pre-event activity, 0.01 was used to calculate the relative change).

The temporal impact of dendritic spikes on somatic activity was analyzed in a shorter (bins: 1 sec, ±49 bins before and after the event bin) and in a longer (bins: 5 sec, 24 bins before and after the event bin) window around the event, including only those events that were preceded and followed by imaging throughout the analyzed time window. Bin centers were aligned to the time of the spike (as defined above). Inferred spikes were summed in each time bin, and the resulting data points were averaged in three time periods: control activity (before the event), peak (<20 sec after the event; the bin containing the event was included for Ca^2+^ plateaus but excluded for fDI spikes) and sustained activity following the peak period, as shown by shadings on Figs. 4 (A and B) and 6 (D and E). Because Ca^2+^ events occurring on the decay phase of previous events were excluded (see Event detection and analysis), our dataset may be biased to events that were immediately preceded by a silent period. Therefore, the control period for comparison excluded the first 20 s before the event (the approximate duration of plateaus). In addition, we confirmed that, in the same imaging recordings that contained fDI spikes, randomly selected silent imaging frames (no activity at least 2 s away from any suprathreshold event) are not followed by increased activity (fig. S7I).

Most of the events identified as putative dendritic spikes based on the abovementioned criteria were measured during sessions where the mouse navigated in the familiar VCs. Some of the events (*n* = 2 Ca^2+^ plateaus in one cell; *n* = 11 fDI spikes in a different cell) occurred in occasional sessions where the animal was also exposed to a novel VC. None of the two Ca^2+^ plateaus and ^3^/_11_ of the fDI spikes in novel VCs induced increased activity using the above criteria. The data from familiar and novel VCs were pooled.

To analyze the distance of plateaus and fDI spikes relative to the RZ, we considered the distance range around the RZ that overlaps across all three VCs (from −35 to +15 bins) and examined whether the observed frequency of events in these areas was different from that expected by random occurrence.

### Analysis of spatial tuning

While most CA3PCs produced at least 1 Ca^2+^ transient over the course of the imaging sessions, we considered cells to be “active” with at least 5 Ca^2+^ events per 10 min. Spatial tuning was only analyzed in these active cells. To estimate the underlying spiking activity for evaluating spatial tuning, the baseline-corrected fluorescence traces were deconvolved using OASIS (*70*) with tau = 0.6 s (*71*). Inferred spikes on parts of the baseline-corrected raw fluorescence trace below four times the noise SD (see Ca^2+^ data pre-processing) for the given ROI were removed. We restricted our analysis to periods when the animal was running (velocity of >5 cm/s). The spikes inferred from Ca^2+^ activity were spatially binned (75 bins), averaged across the three neighboring spatial bins, summed across laps, and then the total spike count was divided by the time spent in each bin to estimate the position-dependent activity rate (“tuning curve”) of individual cells. Tuning curves were calculated separately in the two corridors.

In dual-plane sessions (when the frame rate was relatively low), the animal could occasionally move multiple spatial bins between two subsequent imaging frames. In this case, the spikes inferred at a given frame were distributed equally among all the spatial bins covered since the preceding imaging frames within the same corridor. This procedure was not done when the animal was teleported to the start of the next VC; therefore, some spatial bins at the end of the corridors had missing data due to the low frame rate (black bins in Figs. 4, D and F, and 6C and fig. S4, E and G).

We computed several metrics to identify spatially tuned cells in our recordings, similarly to that described previously (*68*). Specifically, spatial tuning was evaluated by calculating three different parameters: (i) spatial reliability, i.e., the average Pearson correlation between the spiking in individual laps and the neuron’s tuning curve; (ii) tuning specificity, i.e., the SD of the tuning curve divided by the length of the corridor; and (iii) spatial information content, i.e., the estimated mutual information between position and the tuning curve (*72*). Significance of the spatial tuning and corridor selectivity of the cells was identified by bootstrapping, where we created 1000 shuffled rate maps by circularly shifting the spiking activity of each cell relative to the true position of the mouse and then dividing it into five chunks of at least 500 frames whose order was randomly permuted. Cells with significantly higher values in at least one of the spatial metrics or selectivity (*P* < 0.05) were considered as spatially tuned in the given corridor. We used the Holm-Bonferroni method to correct for multiple statistical tests.

### Analysis of PF formation by Ca^2+^ plateaus

PF formation by dendritic Ca^2+^ plateaus (BTSP) is characterized by robust somatic burst activity during PF formation, appearing as stronger activity in the formation lap than in subsequent laps (*14*, *34*, *37*). In CA1PCs, BTSP-induced PF formation is also characterized by a backward shift in the PF center of mass (COM) due to the asymmetric temporal coincidence kernel of synaptic activity and plateau potential for synaptic potentiation. This backward COM shift by plateau-induced PF formation has been recently reported to be weaker or absent in CA3PCs (*43*, *73*). To characterize PF formation induced by slow Ca^2+^ plateaus, we determined the ratio of activity during the formation lap and subsequent laps (“gain”) and the shift of the PF COM after PF formation similar to previous studies (*36*, *39*). We first calculated the somatic spike rate matrix (SSRM) of a given cell by dividing the total number of spikes in each spatial bin and each lap by the amount of time spent in that given spatial bin in that given lap

$$\text{SSR}M_{\mathrm{ij}}=\frac{s_{\mathrm{ij}}}{t_{\mathrm{ij}}}$$

We then calculated the maximum rate in each lap across all spatial bins. In this analysis, those laps were considered to be active laps where the maximum activity within the lap is greater than 10% of the maximum activity across all laps.

The lap where the Ca^2+^ plateau occurred was considered as the reference lap. The maximum spike rate across all spatial bins (*b*) within the reference lap was termed the reference maximum rate. Next, in each of the five subsequent active laps, we calculated the same value, calling them subsequent maximum rates. We then calculated the ratio of the reference maximum rate to each subsequent maximum rate. Gain is defined as the average of these ratios

$$G=\frac{1}{5}\sum_{\text{lap}=1}^{5}\frac{\underset{b}{\text{max}}\text{SSR}M_{\text{ref}}}{\underset{b}{\text{max}}\text{SSR}M_{\text{ref}+\text{lap}}}$$

To calculate the shift, we first calculated the COM of the spike rates in the reference lap across all the spatial bins that fall within the detected boundaries of the PF, which we call reference COM. We then calculated a similar spike rate COM of the five subsequent active laps and calculated the difference of each from the reference COM. Shift is then defined as the average of these differences

$$S=\frac{1}{5}\sum_{\text{lap}=1}^{5}\text{CO}M_{\text{ref}+\text{lap}}-\text{CO}M_{\text{ref}}$$

### Histology

For post hoc analysis, mice were anesthetized with isoflurane and perfused transcardially with ice-cold Ringer solution (Fresenius Kabi). Extracted brains were postfixed in 4% paraformaldehyde (PFA; Molar Chemicals) overnight at 4°C. Next day, the brains were removed from PFA and rinsed three times for 10 min in phosphate buffer (PB). Horizontal slices (60 μm thick) were cut on a vibratome (Leica VT1000 S). Slices were additionally washed three times with PB for 10 min and labeled for 4′,6-diamidino-2-phenylindole (DAPI; Thermo Fisher Scientific). Mounted slices were covered with Vectashield Antifade Mounting Medium (Vector Laboratories). Confocal images were acquired on a Nikon Ni C2 microscope equipped with a 10× (NA 0.3) and 20× (NA 0.75) objective using 488-nm laser line for GCaMP8s and 405-nm line for DAPI.

### Statistics

Statistical analysis was performed using the Statistica software (version 14.0.1.25, Tibco). Box plots in all figures represent the median (central line), 25th and 75th percentiles (box), and 10th and 90th percentiles (whiskers) of the data. All statistical tests were two tailed. In all figures, statistical significance is indicated as *P < 0.05, **P < 0.01, and ***P < 0.001.
