# Supplementary material for: Distinct types of dendritic Ca2+ spikes in CA3 pyramidal neurons associated with neuronal activity changes during navigation
Source: Sci Adv. 2026 Aug 14;12(33):eaec6015. doi: 10.1126/sciadv.aec6015 (PMC13475495; doi:10.1126/sciadv.aec6015)
Supplement: Supplementary file 1 [file sciadv.aec6015_sm.pdf]

Science Advances
AAAS

# Supplementary Materials for

## Distinct types of dendritic $Ca^{2+}$ spikes in CA3 pyramidal neurons associated with neuronal activity changes during navigation

Snezana Raus Balind *et al.*

Corresponding author: Judit K. Makara, makara.judit@koki.hun-ren.hu

*Sci. Adv.* **12**, eaec6015 (2026)
DOI: 10.1126/sciadv.aec6015

**This PDF file includes:**

Figs. S1 to S8

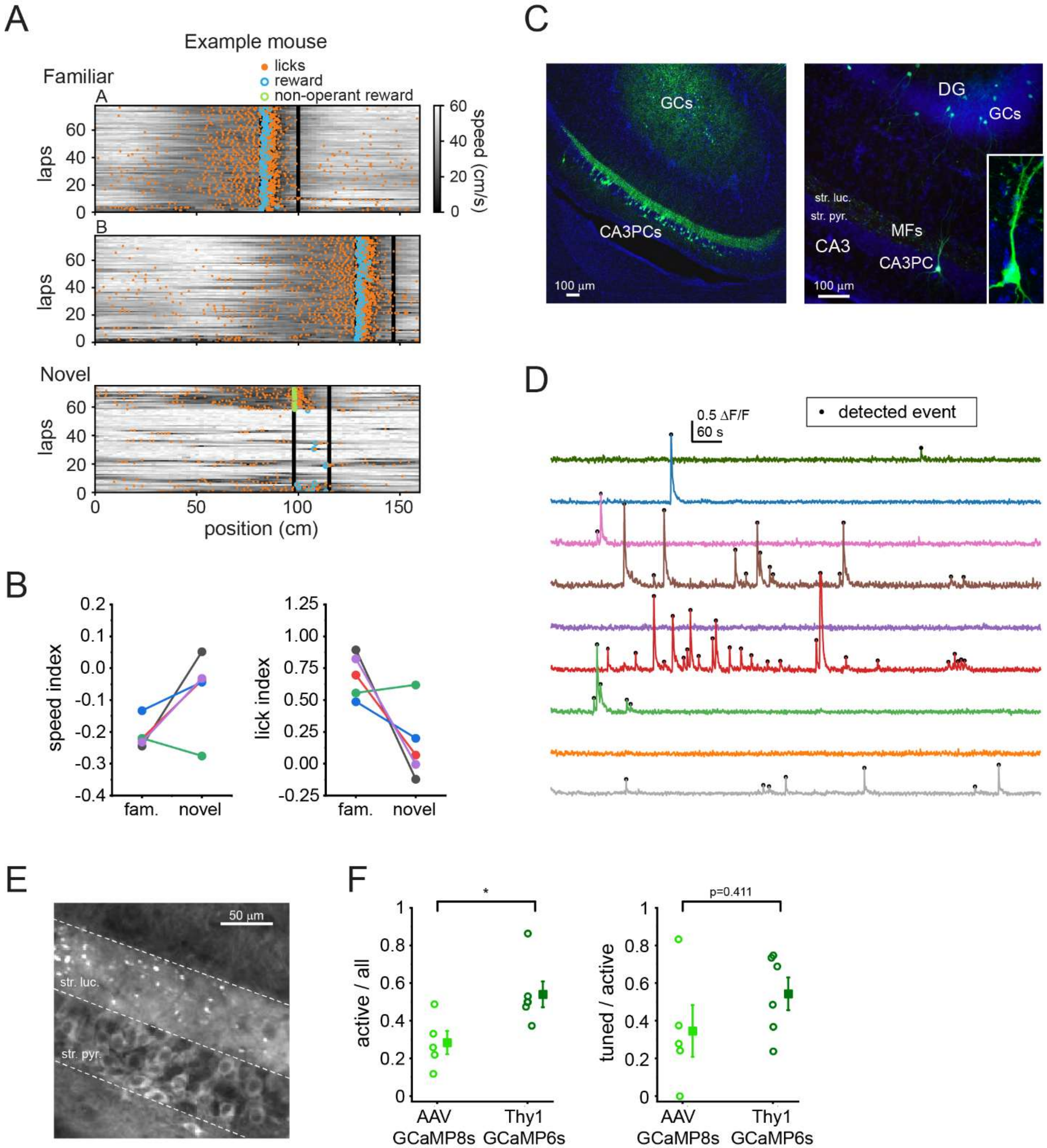


**Supplementary Fig. 1. Behavior in a novel corridor and additional results with dense imaging.**
(**A**) Lap-by-lap speed and lick patterns of a mouse in a session where, after performing the task in the familiar VCs, the animal was exposed to a novel VC (see Fig. 1B). While first the behavior was disrupted, after receiving non-operant reward the animal quickly developed anticipatory behavior before the new RZ.
(**B**) Speed index (left) and lick index (right) in the last 10-10 laps of the two corridors of the familiar VC (fam, mean of the two corridors) and in the first 10 laps of a novel VC in 5 mice. Four mice could not find the new RZ in the novel VC in the first session, whereas one mouse quickly learned the new task.
(**C**) Left, confocal image of a horizontal hippocampal section prepared from a mouse where densely labelled CA3PCs were imaged *in vivo*. Staining was performed for DAPI. Right, fluorescent image of a horizontal

hippocampal section stained for DAPI (blue) *post hoc* from a mouse where sparsely labelled CA3PCs (green) were imaged *in vivo*. Inset shows confocal image of the CA3PC. Note the dense thorny excrescences on the apical dendrite of the CA3PC, labelled mossy fibers in CA3 str. lucidum and granule cells in DG.
(**D**) Detected $Ca^{2+}$ events (black dots) in a representative imaging session with dense labeling.
(**E**) Example FOV of the distal CA3 area in a Thy1-GCaMP6s mouse (mean image of time series, cropped). Approximate borders of str. pyramidale and str. lucidum are indicated by dashed lines.
(**F**) Ratio of active cells (with >5 events/10 min) of all labelled cells (left; Mann-Whitney test p=0.022), and ratio of tuned cells of all active cells (right; Mann-Whitney test p=0.411) in AAV-injected GCaMP8s expressing CA3PCs (light green, n=5 mice, 2-6 sessions averaged per mouse) and in GCaMP6s-expressing CA3PCs in Thy1-GCaMP6s transgenic animals (n=6 mice, 1 or 2 averaged sessions per mouse). Note that the background mouse strains are also different between the two experiments (FVB/AntJ vs C57BL/6J).

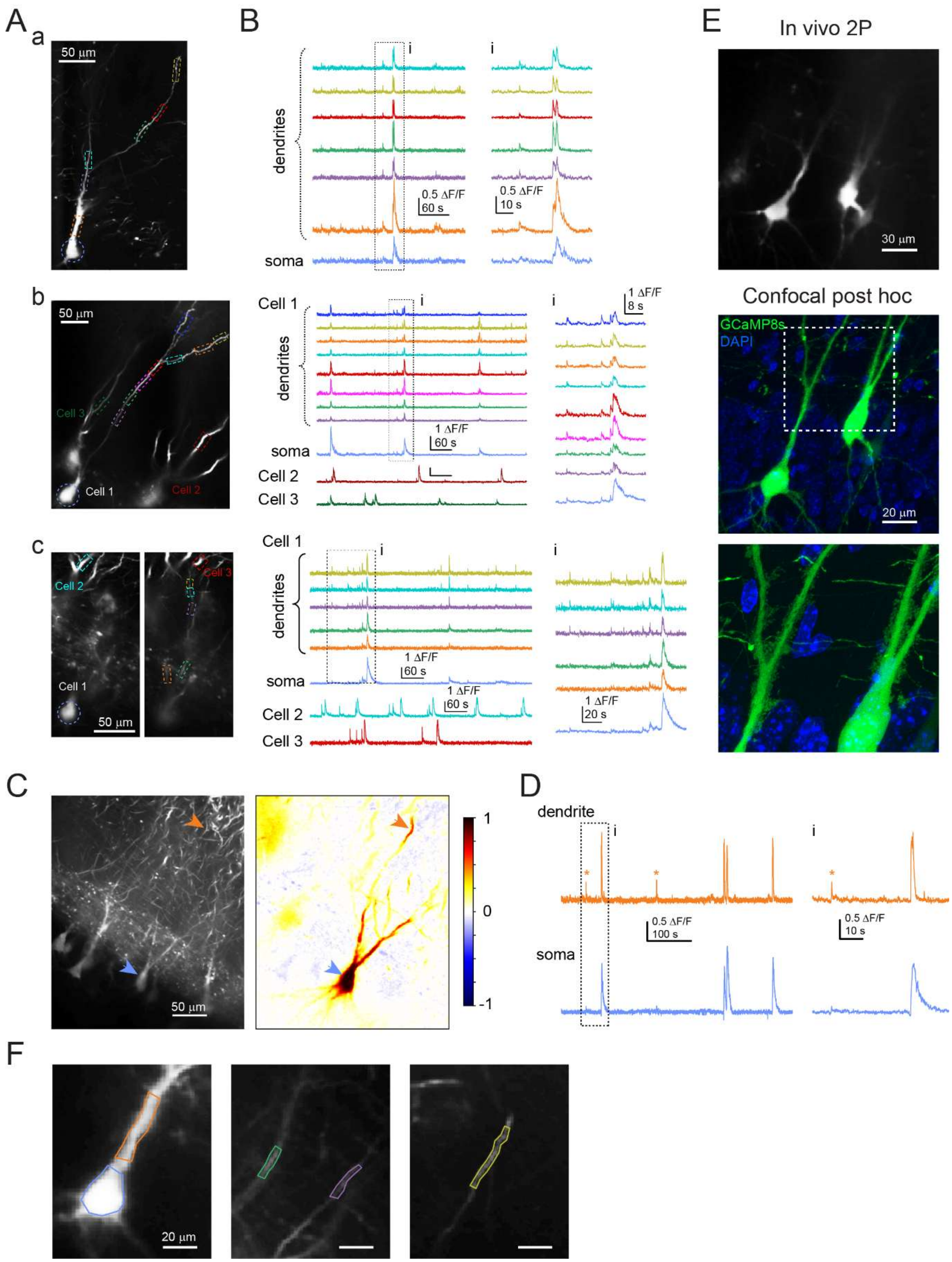


**Supplementary Fig. 2.** ***In vivo*** **imaging in mouse CA3PCs**

(**A**) Examples of 2P imaging from soma and dendrites of three CA3PCs (a-c; mean image of time series, cropped from FOV). Selected ROIs are indicated by colors.

(**B**) Activity of the ROIs indicated in A (corresponding color code). Activity in the dashed box (i) is shown at higher temporal resolution on the right. Note the independent activity of nearby dendrites belonging to other neurons in b and c.
(**C**) 2P $Ca^{2+}$ recording in a mouse with relatively dense GCaMP8s labeling. Left, the soma and a distal apical dendrite of a CA3PC are indicated by blue and orange arrowheads, respectively. Right, correlation of the activity of every pixel in the FOV with the activity of the somatic ROI.
(**D**) Activity of the soma and dendritic segment indicated by the arrowheads in C. Activity in the dashed box (i) is shown at higher temporal resolution on the right. Note the clear soma-dendrite coupling of activity, and the occurrence of some $Ca^{2+}$ events (asterisks) that have large dendritic and small corresponding somatic amplitude.
(**E**) Top, collapsed *in vivo* resonant 2P z-stack including two neurons. Middle, same cells visualized with confocal microscopy after staining for DAPI. Bottom, zoom-in of the image section indicated by dashed box in the middle panel. Note the numerous thorny excrescences on apical dendrites of both neurons.
(**F**) ROIs drawn around neuronal compartments of the CA3PC in Fig. 2. Scale bars, 20 μm.

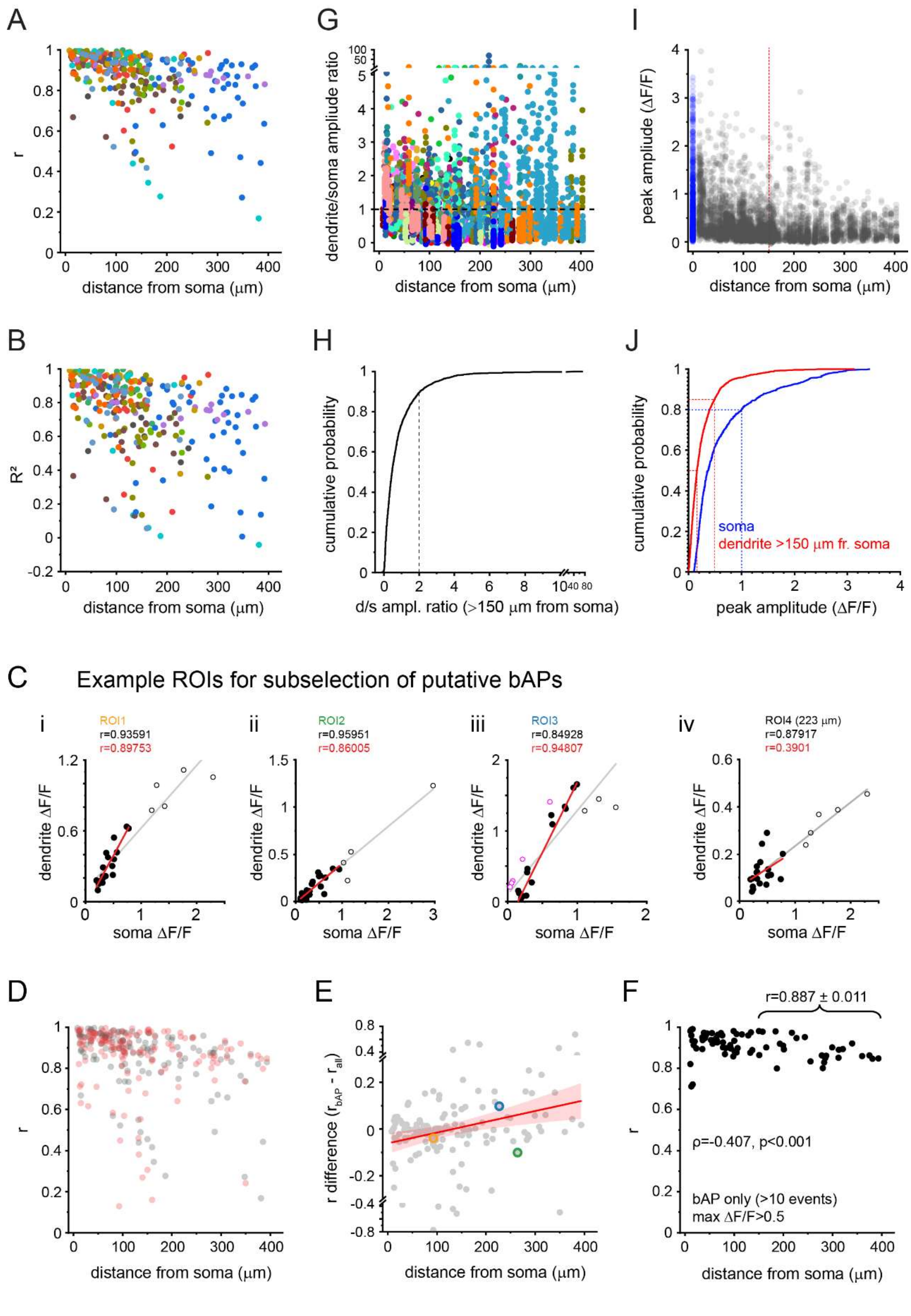


**Supplementary Fig. 3. Characterization of somatodendritic $Ca^{2+}$ events**

(**A**) Pearson's correlation coefficient for dendrite-soma ROI pair $Ca^{2+}$ signal amplitudes as a function of the distance of the dendritic ROI from the soma (same data as in Fig. 2F), color-coded for each cell.

(**B**) Adjusted $R^2$ values of the correlations in A.
(**C-F**) To investigate distance-dependent soma-dendrite $Ca^{2+}$ event amplitude correlations of putative backpropagating APs in our ROIs, we subselected bAPs by removing all putative non-bAP events, i.e. identified fDI events (see Figs. 5-6), identified $Ca^{2+}$ plateaus (see Figs. 3-4) and all remaining events that had somatic amplitudes larger than 1 ΔF/F (potentially reflecting plateaus or longer bursts, especially in recordings without distal ROIs). The remaining events likely included both single APs and short bursts of a few APs.
(**C**) Correlation plots of $Ca^{2+}$ event amplitudes in the dendrite and the soma for example ROIs from several cells. We analyzed putative bAP events only (black filled circles) by removing fDI spikes (magenta open circles) as well as plateaus and other events with larger than 1 ΔF/F somatic amplitude (pooled, black open circles). Correlation with all events included (grey lines) and with only bAPs (red lines) are indicated. Note that r decreased in the ROIs shown in i and ii but increased in the ROI shown in iii. In some ROIs the subselected putative bAP events had very small amplitudes in the dendrite (iv) rendering the measurement of correlation unreliable; such ROIs (maximum dendritic amplitude <0.3 ΔF/F or mean amplitude <0.1 ΔF/F) were excluded from the analysis in D-E.
(**D**) Correlation coefficients for dendrite-soma $Ca^{2+}$ signal amplitudes as a function of the distance of the dendritic ROI from the soma, with all events (grey, $r_{all}$) and with only bAPs (red, $r_{bAP}$). ROIs with maximum event amplitude <0.3 ΔF/F and mean event amplitude <0.1 ΔF/F were ignored for this analysis, as correlation of small events could not be reliably measured.
(**E**) Difference in r ($r_{bAP}$ - $r_{all}$) from D. Red line and shading represents linear fit with confidence intervals; orange, green and blue circles indicate the ROIs in Ci,ii,iii. We found a positive correlation (Spearman ρ=0.234, p=0.004, n=143) so that distal ROIs tended to have higher soma-dendrite amplitude correlations after subselection for bAPs, in line with our conclusion that distal dendrites are more independent from the soma than proximal dendrites.
(**F**) To obtain the best possible estimate of event amplitude correlation of only bAP events, we restricted our examination to those dendritic ROIs where >10 events occurred and where dendritic event amplitudes of bAPs varied in a relatively large range (maximum amplitude >0.5 ΔF/F). In these cases, event amplitude correlations still decreased with distance from soma (Spearman ρ=-0.407, p<0.001, n=66), nevertheless, the correlation was quite high even at >150 µm.
(**G**) Ratio of $Ca^{2+}$ event peak amplitudes in the apical dendrites vs the soma (same data as shown binned in Fig. 2H), color-coded for each cell (color code does not correspond to that in A-B).
(**H**) Cumulative probability of dendrite/soma event amplitude ratios in dendritic ROIs that were located >150 µm distance from the soma (n=2262). 10.43% of events had an amplitude ratio>2 (dashed line).
(**I**) Peak ΔF/F amplitudes corresponding to $Ca^{2+}$ events that were detected in at least one ROI (soma and/or apical dendrites), measured in all imaged ROIs. Only those ROI pairs with ΔF/F>0.1 in at least one of the two compartments were included. N=5364 values corresponding to 884 events in 31 neurons. Somatic event amplitudes are shown in blue, red dashed line indicates 150 µm dendritic distance from the soma.
(**J**) Cumulative probability of somatic (blue, n=963) and distal apical dendritic (>150 µm from soma, red, n=2262) $Ca^{2+}$ signal amplitudes corresponding to events that were detected in any imaged ROI of the cell. Dashed lines represent $80^{th}$ percentile (soma) and $50^{th}$ and $85^{th}$ percentiles (distal apical dendrites).

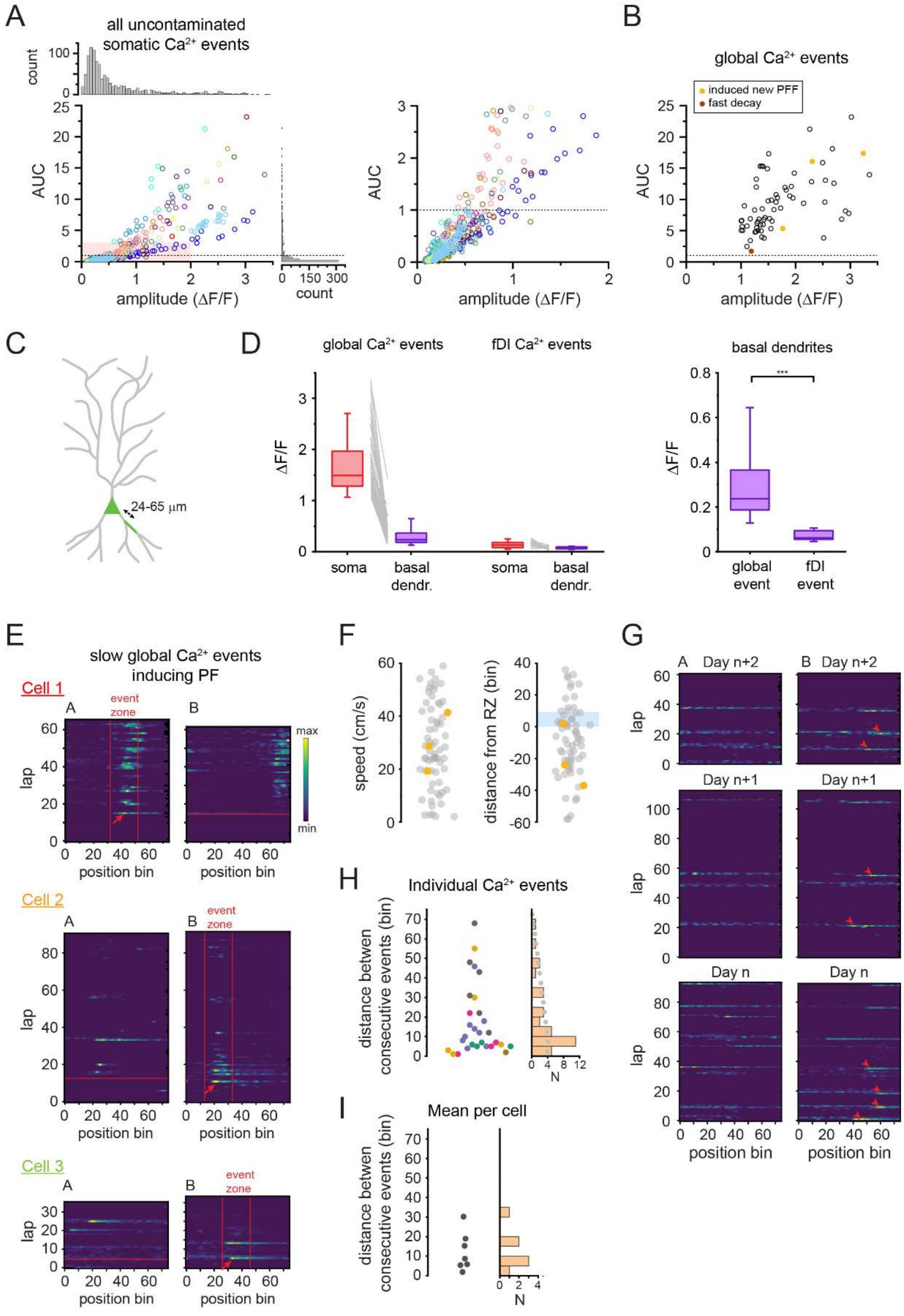


**Supplementary Fig. 4. Characteristics of slow global $Ca^{2+}$ events**

(**A**) Left, peak amplitude vs AUC of individual somatic $Ca^{2+}$ transients (subgroup of events that were not contaminated by major subsequent events before returning to baseline). Each circle represents a $Ca^{2+}$ event,

color coded by mice. AUC=1 (dotted line) is the approximate border separating a population of events with small AUC values from the rest of the events (histogram). Pink shading indicates the region expanded on the right. Note that in the AUC<1 and peak ΔF/F<1 sector, the amplitude and AUC of events have largely linear correlation, while events with AUC≥1 are more heterogeneous, suggesting nonlinear increase in their duration.
(**B**) Somatic peak amplitude and AUC of $Ca^{2+}$ plateaus (circles, n=68) identified as in Fig. 3A (see also Methods). Some of these events were contaminated by subsequent $Ca^{2+}$ transient(s), and therefore are not included in panel A. AUC=1 is indicated by dotted line. Brown symbol indicates an event with fast decay (see Fig. 3B inset). Orange symbols indicate the $Ca^{2+}$ plateaus that induced new place field formation (Fig. 4E-G, I-J, fig. S4E).
(**C**) Schematic showing imaging locations in basal dendrites.
(**D**) Left, comparison of $Ca^{2+}$ signal amplitudes in somatic and basal dendritic ROIs during $Ca^{2+}$ plateaus and fDI spikes. Grey lines represent individual soma-basal dendrite ROI pairs; box plots show median (central line), 25$^{th}$ and 75$^{th}$ percentiles (box) and 10$^{th}$ and 90$^{th}$ percentiles (whiskers) of the data ($Ca^{2+}$ plateaus: n=39 events in 15 cells; fDI spikes: n=22 events in 6 cells). Right panel compares the amplitudes of the two event types in basal dendrites at higher magnification (box plots same as on left panel, p<0.001, Mann-Whitney test).
(**E**) Position-dependent lap-by-lap activity in the two VCs of all three cells where a $Ca^{2+}$ plateau induced a new PF (cell 1 is also shown in Fig. 4E). Red arrows indicate the $Ca^{2+}$ plateaus. Red vertical lines indicate the spatial zone (±10 spatial bins around the event) where formation of a new PF was investigated. Red horizontal line in the other VC indicates the time of the interleaved lap where the $Ca^{2+}$ plateau occurred.
(**F**) Running speed (left) and position relative to the next RZ (right, RZ indicated by blue shadow) of the mice in the spatial bins where $Ca^{2+}$ plateaus (dots) occurred. Orange symbols indicate the $Ca^{2+}$ plateaus that induced place field formation (Fig. 4E-G, I-J, fig. S4E).
(**G**) Lap-by-lap activity of an example CA3PC imaged during three consecutive days. Red arrowheads indicate $Ca^{2+}$ plateaus. Note that the $Ca^{2+}$ plateaus occur at similar locations in corridor B.
(**H**) Left, distance (measured as spatial bins) between the locations of consecutive $Ca^{2+}$ plateaus occurring in the same cells within the same VCs. Analysis included those cells where multiple $Ca^{2+}$ plateaus occurred in the same VC (n=34, events in both VCs and in the same or in different sessions pooled; cells are color coded). Right, histogram of pooled inter-plateau position distances. Grey dots represent expected inter-plateau distance frequencies from random spatial distribution of $Ca^{2+}$ plateaus.
(**I**) Data in H, averaged in each cell (n=7).

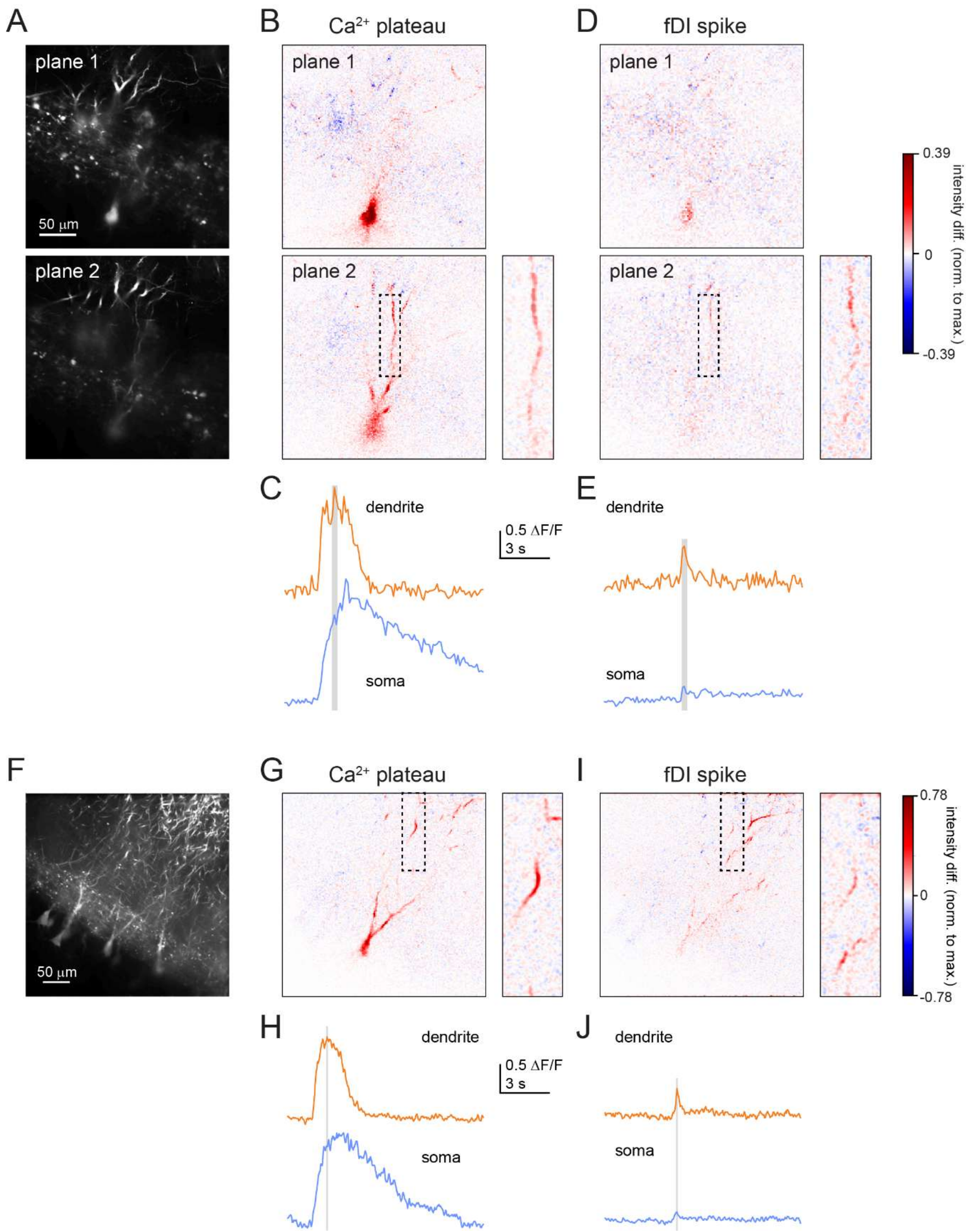


**Supplementary Fig. 5. Representative spatial footprints of dendritic spikes**
(**A**) Example recording with two imaging planes (same cell shown in fig. S2Ac)
(**B**) Difference in fluorescence intensity in the FOV of the cell shown in A, calculated during and before a $Ca^{2+}$ plateau. The spatial footprint demonstrates strong global somatodendritic activity in the neuron. A distal dendrite in dashed box is magnified on the right.

(**C**) $Ca^{2+}$ transient corresponding to the event analyzed in B. Grey shading indicates the three frames during the event that were averaged for the spatial footprint analysis.
(**D-E**) Same as B-C for an fDI spike. Note that the fluorescence difference occurred in the same dendritic segment in B and D.
(**F-J**) Same as A-E for a recording with single-plane imaging and more dense labeling (same cell shown in fig. S2C).
Color scales are normalized to the maximum values in B and G, respectively; ends are clipped.

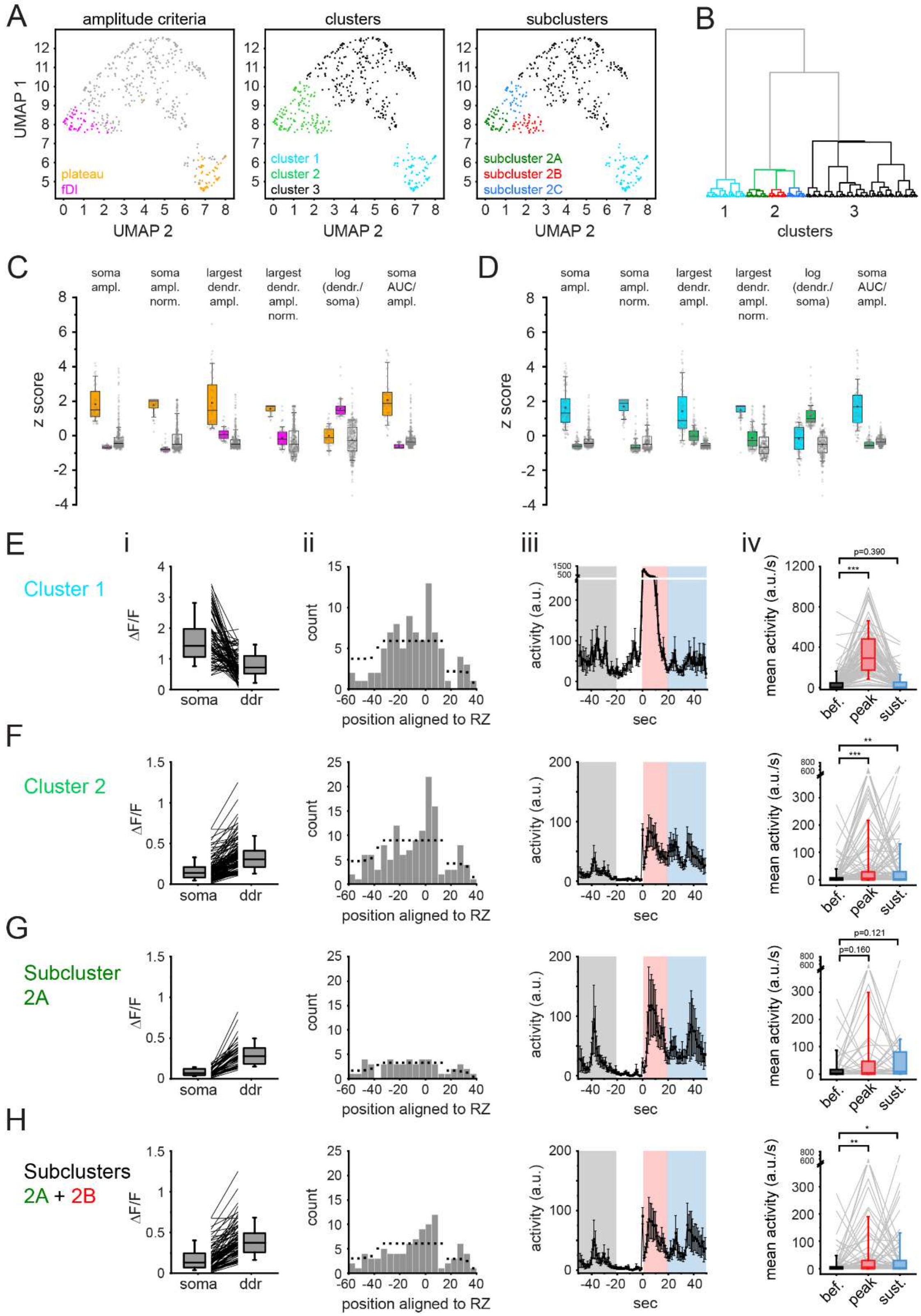

**Supplementary Fig. 6. Clustering of events using UMAP analysis.**

(**A**) Two-dimensional representation of a subgroup of individual events (n=478 events from 25 cells with at least one dendritic segment ≥150 μm from the soma) where all amplitude and kinetic parameters (see C-

D) could be measured. Left, mapping of plateau (orange) and fDI spike (magenta) events (as defined based on amplitude criteria) on the UMAP representation. Middle, three clusters of events defined by Ward hierarchical clustering performed on the UMAP dataset. Right, three subclusters of cluster 2 defined by hierarchical clustering. Note that almost all amplitude-defined plateau events overlap with cluster 1 (except for the only fast event, see Fig. 3B), and amplitude-defined fDI events map onto cluster 2 (subclusters A and to some extent B). Nevertheless, we acknowledge that, instead of strict categories, the underlying space of event properties may be multidimensional and the identified dendritic event types likely represent dominant regimes within a broader continuum.

(**B**) Dendrogram produced by hierarchical clustering.

(**C**-**D**) Distribution of the parameters used for UMAP (see Methods). Only those events were included in this analysis that were not contaminated by consecutive events, and thus clean AUC could be measured (color code of events as in A).

(**C**) Parameters shown separately for event groups defined based on amplitude criteria described in the main results (see Figs. 3 and 5).

(**D**) Parameters shown separately for the event groups defined by UMAP-based hierarchical clustering.

(**E**-**H**) Properties and effect of events belonging to different UMAP clusters. (i) Somatic and dendritic event amplitudes; (ii) histogram of the distance of the spatial bins where the events occurred from the RZ start bin (all VCs pooled), and expected frequency of events occurring randomly (black symbols); (iii) somatic activity (inferred spikes) in a ±50 seconds time window before and after the event (shadings as in Figs. 4A and 6D; for analysis of cluster 2 (F-H) the event bin was excluded); (iv) summary of mean somatic activity in the time periods indicated in iii. Friedman test, E: $p<0.001$, F: $p=0.007$, G: $p=0.508$, H: $p=0.044$; individual Wilcoxon test results are indicated on the panels.

Somatic activity after cluster 1 and cluster 2 events were qualitatively similar to that following amplitude-defined plateaus and fDI spikes, respectively (Fig. 4A, Fig. 6D). We also note that none of the additional cluster 1 events (that were not included to plateaus) induced a new PF.

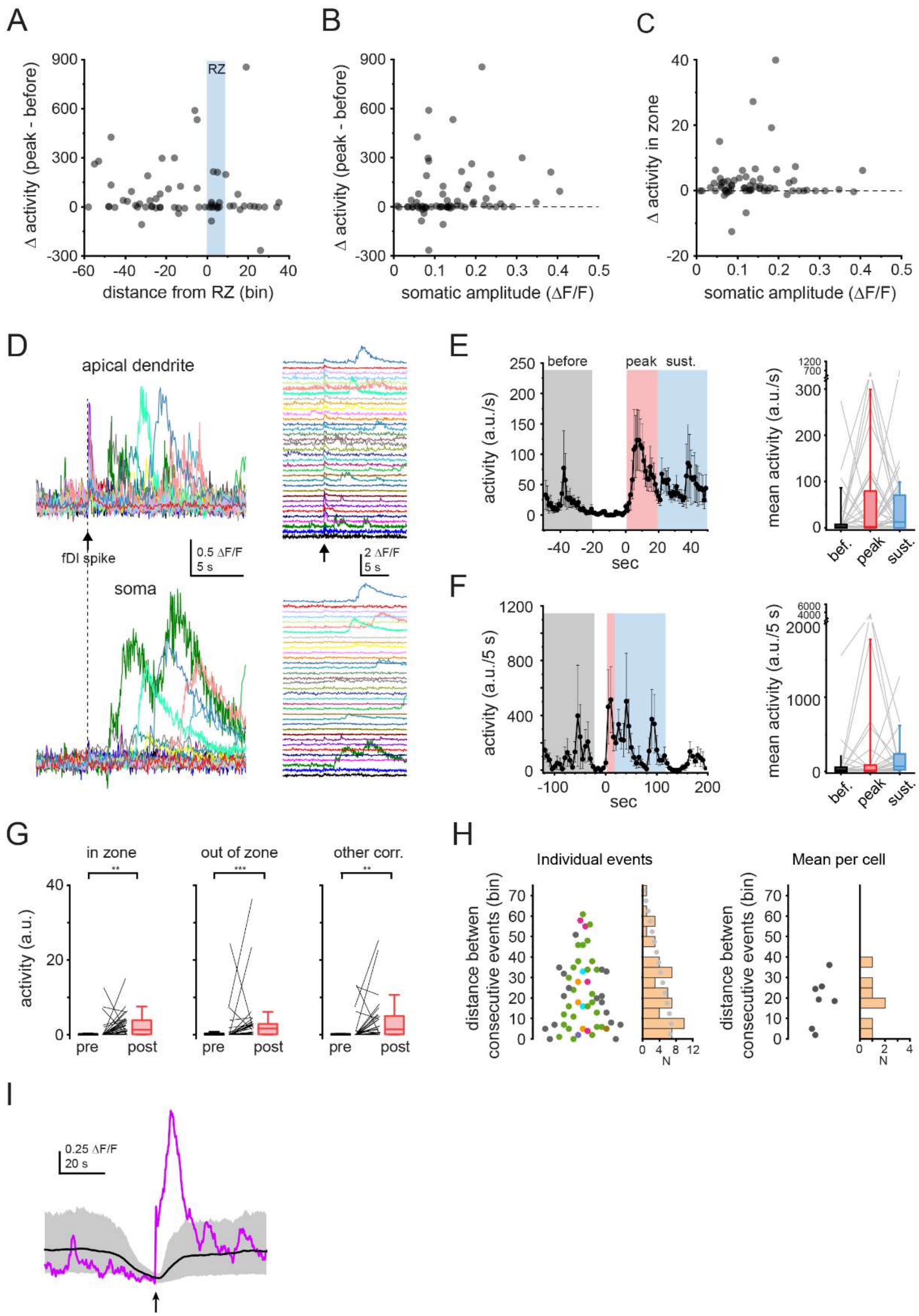


**Supplementary Fig. 7. Additional characterization of fDI spikes.**

(**A**) Change in somatic activity in the first 20 seconds after fDI spikes, as a function of the distance of the spatial bin of fDI spike occurrence from the RZ. No difference was found between the effect of fDI spikes occurring before or in the RZ (bins -35 to +9) and those occurring after the RZ (bins +10-35). Mann-Whitney test: p=0.561; n=44 before/in RZ, n=13 after RZ.

(**B**) Change in somatic activity in the first 20 seconds after the fDI spike, as a function of the corresponding somatic $Ca^{2+}$ signal amplitude. While there was a positive correlation between somatic fDI spike $Ca^{2+}$ signal amplitude and the change in somatic activity (Spearman R=0.408, p<0.001, n=68), even fDI spike with <0.2 ΔF/F somatic $Ca^{2+}$ signal amplitude had a significant effect (one-sample Wilcoxon test vs 0, p=0.001, n=53).
(**C**) Change in somatic activity in the event zone of the fDI spike, as a function of the corresponding somatic $Ca^{2+}$ signal amplitude. No correlation was found between somatic fDI spike $Ca^{2+}$ signal amplitude and change in in-zone activity (Spearman R=-0.012, p=0.922, n=63).
(**D**) Selection of fDI spikes with somatic event amplitude below the event detection threshold (noise +4 SD). Individual dendritic (top) and somatic (bottom) fDI spike-related $Ca^{2+}$ signals aligned to their start point (n=35 events in 6 cells; events where initial trunk was imaged instead of soma are not shown). Traces are displayed separately on the right.
(**E-G**) Analysis of the impact of fDI spikes with somatic event amplitude below the event detection threshold.
(**E**) Left, somatic activity (inferred spikes) in a ±50 seconds time window before and after fDI $Ca^{2+}$ spikes with undetected somatic events. Analysis as in Fig. 6D; the event bin was excluded from analysis. Right, summary of mean somatic activity in the time periods indicated by shadings on the left (n=39 events with imaging in the analyzed period). Friedman test, p=0.175; Wilcoxon test ctr vs 0-20: p=0.058; ctr vs 20-50: p=0.063.
(**F**) Same as in E but in a wider time window and using 5 sec time bins (n=26 events with imaging in the analyzed period). Analysis as in Fig. 6E; the event bin was excluded from analysis. Friedman test, p=0.050; Wilcoxon test ctr vs 0-20: p=0.567; ctr vs 20-50: p=0.031.
(**G**) Comparison of mean somatic activity/bin measured before (last 3-5 laps) and after (until lap 5-10) the fDI $Ca^{2+}$ spike with undetected somatic events, calculated in-zone (left, p=0.001), out-of-zone in the same VC (middle, p<0.001) and in the other VC (right, p=0.003). Lines represent individual fDI spikes. Box plots show the median (central line), $25^{th}$ and $75^{th}$ percentiles (box) and $10^{th}$ and $90^{th}$ percentiles (whiskers) of the data (n=36 events); Wilcoxon tests.
(**H**) Left, distance (measured as spatial bins) between the locations of consecutive fDI spikes occurring in the same cells within the same VCs. Analysis included those cells where multiple fDI spikes occurred in the same VC (n=57, events in both VCs and in the same or in different sessions pooled; cells are color coded). Histogram on the right shows pooled inter-spike position distances; grey dots represent expected inter-spike distance frequencies from random spatial distribution of fDI spikes.
Right, data averaged in each cell (n=7).
(**I**) Randomly selected silent time points (in the same imaging recordings that contained fDI spikes) are not followed by increased activity. Magenta: mean ΔF/F trace of fDI spike events. Black line and grey shading: mean and 95% confidence interval of ΔF/F traces 50 seconds before and after 1000 randomly selected imaging frames for each fDI spike, at least 2 seconds away from any suprathreshold activity (baseline mean + 4 SD). Arrow points to the fDI spike.

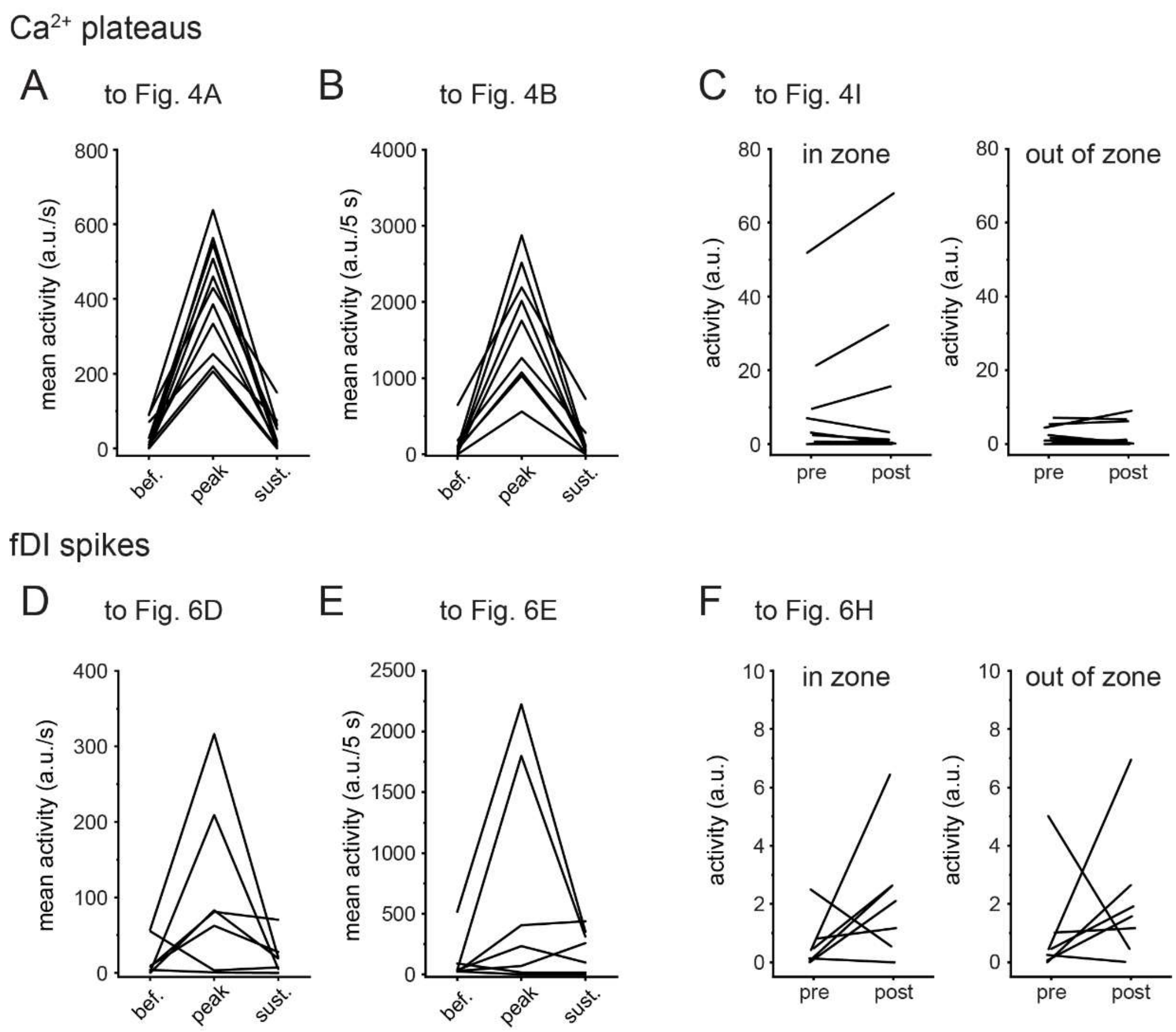


**Supplementary Fig. 8. Temporal and spatial effects of $Ca^{2+}$ plateaus (A-C) and fDI spikes (D-F) on neuronal activity, data pooled per animal**

Data shown in the corresponding main figures, with events averaged in each animal. Statistical analysis:

(**A**) Friedman test: $p<0.001$, Wilcoxon tests: bef. vs peak: $p=0.003$, bef. vs sust: $p=0.959$, n=11 mice.

(**B**) Friedman test: $p=0.001$, Wilcoxon tests: bef. vs peak: $p=0.007$, bef. vs sust: $p=0.593$, n=9 mice.

(**C**) In zone: Wilcoxon test: $p=0.444$, Out of zone: $p=0.878$, n=11 mice.

(**D**) Friedman test: $p=0.180$, Wilcoxon tests: bef. vs peak: $p=0.062$, bef. vs sust: $p=0.735$, n=7 mice.

(**E**) Friedman test: $p=0.367$, Wilcoxon tests: bef. vs peak: $p=0.090$, bef. vs sust: $p=0.310$, n=7 mice.

(**F**) In zone: Wilcoxon test: $p=0.090$, Out of zone: $p=0.310$, n=7 mice.
